# Transcriptomic analysis of transgressive segregants revealed the central role of photosynthetic capacity and efficiency in biomass accumulation in sugarcane

**DOI:** 10.1038/s41598-018-22798-5

**Published:** 2018-03-13

**Authors:** Ratnesh Singh, Tyler Jones, Ching Man Wai, John Jifon, Chifumi Nagai, Ray Ming, Qingyi Yu

**Affiliations:** 1 Texas A&M AgriLife Research Center at Dallas, Texas A&M University System, Dallas, TX 75252 USA; 2 Hawaii Agriculture Research Center, Kunia, HI 96759 USA; 3 Department of Plant Biology, University of Illinois at Urbana-Champaign, Urbana, IL 61801 USA; 4 Texas A&M AgriLife Research Center at Weslaco, Texas A&M University System, Weslaco, TX 78596 USA; 5 Center for Genomics and Biotechnology, Fujian Provincial Key laboratory of Haixia applied plant systems biology, Haixia Institute of Science and Technology, Fujian Agriculture and Forestry University, Fuzhou, Fujian Province China; 6 Department of Plant Pathology & Microbiology, Texas A&M University, College Station, TX 77843 USA

## Abstract

Sugarcane is among the most efficient crops in converting solar energy into chemical energy. However, due to its complex genome structure and inheritance, the genetic and molecular basis of biomass yield in sugarcane is still largely unknown. We created an F2 segregating population by crossing *S*. *officinarum* and *S*. *spontaneum* and evaluated the biomass yield of the F2 individuals. The F2 individuals exhibited clear transgressive segregation in biomass yield. We sequenced transcriptomes of source and sink tissues from 12 selected extreme segregants to explore the molecular basis of high biomass yield for future breeding of high-yielding energy canes. Among the 103,664 assembled unigenes, 10,115 and 728 showed significant differential expression patterns between the two extreme segregating groups in the top visible dewlap leaf and the 9^th^ culm internode, respectively. The most enriched functional categories were photosynthesis and fermentation in the high-biomass and the low-biomass groups, respectively. Our results revealed that high-biomass yield was mainly determined by assimilation of carbon in source tissues. The high-level expression of fermentative genes in the low-biomass group was likely induced by their low-energy status. Group-specific expression alleles which can be applied in the development of new high-yielding energy cane varieties via molecular breeding were identified.

## Introduction

Sugarcane (*Saccharum* spp. Poaceae), the world’s leading biofuel crop, is among the most efficient crops in converting solar energy into chemical energy and has a favorable input/output energy ratio^1,2^. The level of the input/output energy ratio depends on cultural practices and the cropping cycle. It has been reported that the first generation ethanol production sugarcane grown in Brazil under a 12 month crop cycle has an energy balance with a greater than 1:8 energy input/output ratio^3^, while the input/output ratio in Louisiana (~9 month crop cycle), is only 1:3-4^2^, both of which are much more energy efficient than corn at about 1:1.5-3^2^.

Sugarcane belongs to the genus *Saccharum* L. in the Poaceae family. The genus *Saccharum* includes six polyploid species with variable size and number of chromosomes, namely *S*. *spontaneum*, *S*. *robustum*, *S*. *officinarum*, *S*. *barberi*, *S*. *sinense*, and *S*. *edule*^4^. Among these six species, *S*. *spontaneum* (2n = 40 to 128) and *S*. *robustum* (2n = 60, 80, and up to 200) are wild species and the remaining four species, *S*. *officinarum*, *S*. *barberi*, *S*. *sinense*, and *S*. *edule*, are domesticated^5,6^. The initial high sugar content species *S*. *officinarum* (2n = 80, x = 10) was domesticated in New Guinea about 10,000 years ago, likely selected from a high sugar content *S*. *robustum*^7,8^. *S*. *officinarum*, *S*. *barberi*, and *S*. *sinense* have been used for sugar production before modern sugarcane breeding programs via interspecific hybridization started near the end of the 19^th^ century.

The major breakthrough in modern sugarcane breeding was introgression of resistance genes for biotic and abiotic stresses from the wild species *S*. *spontaneum* into the domesticated high-sugar species *S*. *officinarum* by interspecific hybridizations. All current modern sugarcane cultivars are hybrids with 70–80% of the genome from *S*. *officinarum*, 10–20% of the genome from *S*. *spontaneum*, and 10% recombinants^9–11^. The breeding strategy for developing energy cane is similar to that for traditional sugarcane since it involves interspecific crosses to incorporate stress tolerance and high fiber content from *S*. *spontaneum* but differs from sugarcane breeding in that fiber content is preferred.

All species in the *Saccharum* genus are polyploid and there are no related diploid or tetraploid progenitors known. *Saccharum* species have undergone at least two whole genome duplications to become octoploid since their divergence from a common ancestor shared with sorghum about 8 million year ago^12,13^. Since the two wild species *S*. *robustum* (x = 10) and *S*. *spontaneum* (x = 8) have different basic chromosome numbers^5,6,14,15^, the two rounds of duplications might have occurred recently, after speciation separated the two wild species within 2 million years^13^. Although each octoploid has eight genomes, it is difficult to distinguish each individual genome in part because every genome would be a mosaic of all eight genome segments^15–18^, since every chromosome is free to pair and recombine with any one of the other seven homologous chromosomes during meiosis.

Plant biomass yield is a complex trait that is controlled by many external factors (e.g. incident solar radiation, moisture and nutrient supply, etc.) and plant processes such as light interception efficiency, energy conversion efficiency, photosynthetic carbon dioxide assimilation, carbon partitioning efficiency, source-sink balance etc.^19^. In plants with the C4 photosynthetic pathway (or the Hatch-Slack cycle), approximately 6% of incident solar radiation is converted into plant biomass and the rest is lost during light interception, CO_2_ assimilation, carbohydrate synthesis, and respiration^19^. Among these, about 2.5% of the total energy is consumed in respiration^19^. The primary photosynthetic products arise in source tissues (leaves) and are translocated to sink tissues for metabolism and/or storage. In sugarcane, the major sinks include immature leaf rolls, young/expanding leaves, internodes, and root systems. Source and sink metabolism are tightly coupled to avoid imbalances between supply and demand^20–22^. Therefore, metabolism in both the source and sink is important for biomass production. In many plants, including sugarcane, photosynthetic performance in source leaves is regulated by sink strength^21,22^.

In this study, we created a segregating population by crossing *S*. *officinarum* and *S*. *spontaneum*, which contain similar genetic makeup of modern sugarcane and energy cane cultivars. This population exhibited transgressive segregation in biomass yield. We sequenced transcriptomes of both source and sink tissues from extreme segregants to characterize the molecular basis of high-biomass yield from transgressive segregation and potentially facilitate development of high-yielding energy cane varieties.

## Results

### Evaluation of biomass yield of the segregating population

**Figure 1:**
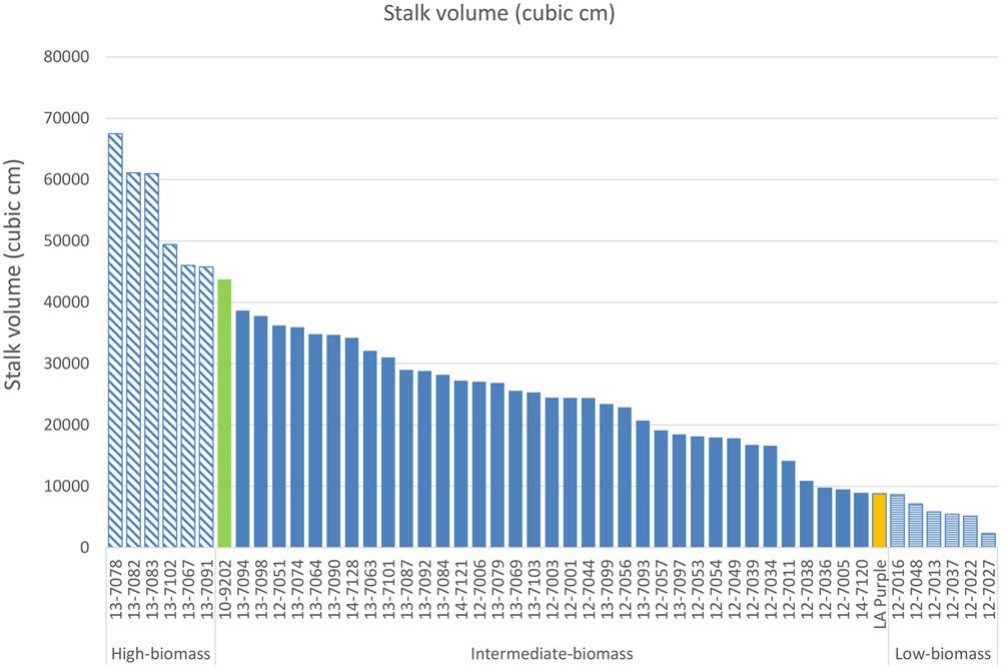
Distribution of the stalk volumes of the *S*. *officinarum* × *S*. *spontaneum* F2 clones.

Field performance of the segregating population was evaluated by assessing stalk volume and dry weight. *Saccharum spontaneum* is listed as a Federal Noxious Weed by USDA-APHIS and is prohibited from field planting. Therefore, the field performance of the parent US56-14-4 could not be evaluated. Stalk volume-related parameters, including stalk diameter, stalk height, and stalk number, were collected when plants were 8.5-month old and used to calculate stalk volume for 47 F2 individuals along with the parent LA Purple (*S*. *officinarum*) and the F1 10-9202. Stalk volume varied over a wide range (29-fold difference) among the F2 individuals. The highest stalk volume was 67,493 cm^3^ and the lowest was 2,306 cm^3^ (Fig. 1, Supplementary Table S1). In comparison with the parent LA Purple and the F1 10-9202, the highest stalk volumes among F2 individuals represented 668% and 54% increases, respectively. Dry weight was collected when plants were 1-year old. A strong correlation between the stalk volumes collected at 8.5-month old and the dry weight collected at 1-year old was observed with the correlation coefficient calculated at 0.86. The highest dry weight of the F2 individuals was 47 kg, representing an increase of 1075% and 30.6% compared to the parent LA Purple and the F1 10-9202, respectively.

### Transcriptome sequencing of the extreme segregants and de novo transcriptome assembly

Based on field evaluation of biomass yield, we selected six F2 individuals whose estimated biomass yield were lower than the parent LA Purple and six clones whose estimated biomass yield were higher than the F1 10-9202 as extreme segregants. The selected clones are shown in Fig. 1. The source tissue, the top visible dewlap leaf, and the sink tissue, the 9^th^ internode, were collected from each selected clone and used for transcriptome sequencing. Transcriptome sequencing of the selected extreme segregants is summarized in Table 1. A total of 44.3 GB Illumina raw sequences in 293.5 million reads were obtained. After quality trimming and removing adapter sequences, 31.6 GB in 231.3 million reads with average of 19.3 million clean reads per sample were obtained (Table 1).

**Table 1:** Summary of transcriptome sequencing of the selected extreme F2 segregants, parents, and the F1 10-9202. L: low-biomass group; H: high-biomass group; P: parent; M: million.

Sample	Leaf				Internode			
	Raw sequence		Trimmed sequence		Raw sequence		Trimmed sequence	
	Read number (M)	Total length (Mb)	Read number (M)	Total length (Mb)	Read number (M)	Total length (Mb)	Read number (M)	Total length (Mb)
7013 (F2-L)	10.79	1,629.08	8.82	1,228.77	1.39	210.31	1.00	130.91
7016 (F2-L)	11.19	1,690.19	9.27	1,298.35	0.45	67.23	0.30	38.14
7022 (F2-L)	8.68	1,310.23	6.71	896.05	2.96	446.94	1.99	253.58
7027 (F2-L)	12.81	1,933.92	10.93	1,555.70	0.33	49.27	0.21	26.19
7037 (F2-L)	9.02	1,361.84	6.98	958.45	0.52	78.85	0.37	47.96
7048 (F2-L)	11.22	1,694.90	9.05	1,257.72	13.33	2,013.36	11.24	1,551.54
7067 (F2-H)	21.24	3,207.89	17.43	2,382.31	44.54	6,725.22	35.27	4,832.95
7078 (F2-H)	13.98	2,111.21	10.42	1,412.25	10.13	1,530.06	8.01	1,095.64
7082 (F2-H)	16.83	2,541.24	12.66	1,700.32	20.83	3,145.26	14.24	1,786.63
7083 (F2-H)	15.07	2,275.24	11.37	1,500.17	14.01	2,114.84	11.86	1,679.34
7091 (F2-H)	20.87	3,150.94	16.70	2,269.77	12.31	1,858.41	9.74	1,355.42
7102 (F2-H)	12.91	1,948.72	10.21	1,409.24	8.04	1,214.78	6.55	912.00
10–9202 (F1)	23.32	3,522.03	17.87	2,410.66	7.63	1,151.49	5.95	813.73
LA-Purple (P)	3.72	560.97	2.64	344.01	4.28	646.44	3.19	432.35
US56–14–4 (P)	10.07	1,521.10	7.85	1,072.89	3.86	583.45	2.76	360.66
**Total**	**201**.**72**	**30**,**459**.**49**	**158**.**90**	**21**,**696**.**65**	**144**.**61**	**21**,**835**.**92**	**112**.**69**	**15**,**317**.**03**
**Parents + F1**	**37**.**11**	**5**,**604**.**10**	**28**.**36**	**3**,**827**.**56**	**15**.**77**	**2**,**381**.**38**	**11**.**90**	**1**,**606**.**74**
**Total F2**	**164**.**61**	**24**,**855**.**39**	**130**.**54**	**17**,**869**.**09**	**128**.**84**	**19**,**454**.**55**	**100**.**78**	**13**,**710**.**30**
**Mean per sample**	**13**.**45**	**2**,**030**.**63**	**10**.**59**	**1**,**446**.**44**	**9**.**64**	**1**,**455**.**73**	**7**.**51**	**1**,**021**.**14**

To obtain a reliable reference assembly for differential gene expression analysis between the two extreme segregating groups, we further sequenced transcriptomes of the top visible dewlap leaf and the 9^th^ internode from the two parents (LA-Purple and US56-14-4) and the F1 10-9202 (Table 1). A total of 40.3 million clean reads of the two parents and the F1 10-9202 were used to create a reference assembly. Assembly of the reference transcriptome yielded 77,221,432 bases distributed in 125,156 transcripts (Supplementary Table S2). These assembled transcripts originated from 103,664 unigenes. The cumulative assembled length of the longest isoforms from each gene accounted for 54,507,395 bases. The average N50 of the reference assembly was 893 and 621 bases for transcripts and unigenes, respectively (Supplementary Table S2).

### Functional annotation of assembled reference transcriptome

Assembled transcripts were annotated using Trinotate pipeline and Mercator web server designed for automatic functional annotation of transcriptomes. Among the assembled sequences, we could annotate 35,378 unigenes (34.13% of the total unigenes) with at least one of the databases in Trinotate pipeline (Table 2). Among the annotated unigenes, the highest proportion (29.76% of the total unigenes) was annotated using BLAST search against the Gene Ontology (GO) reference database. Of the total assembled unigenes, 1.26% and 4.71% were predicted to code proteins with signal peptide and transmembrane topology, respectively. Mercator, a web server for annotation of plant sequences, annotated 28,236 unigenes into 35 functional BINs (Supplementary Fig. S1). Trinotate and Mercator failed to assign 65.87% and 72.76% of total unigenes to a known protein or function, respectively. Among the 61,966 unannotated unigenes, 46.18% showed similarity to Sorghum CDS at e-value cutoff of 1e^−5^. Similarly, 71.57% of unannotated unigenes showed similarity to one or more sequences in NCBI non-redundant database at the e-value cutoff of 1e^−5^. The N50 of unannotated subset of unigenes was 319 bp while the one for annotated fraction was 1218 bp (Supplemental Table S3), which suggested that the failure of annotation might be largely caused by incomplete or fragmented transcriptome assemblies. The annotated fraction of the assembled sugarcane CDS sequences accounts for the 41,698 unigenes, which had detectable homology to 18,604 sorghum genes (56.32% of the total sorghum genes). Since approximately 34% of sorghum genes were unannotated^23^, our annotated sugarcane gene set may represent the majority of the homologous genes of the annotated gene set in sorghum genome.

**Table 2:** Annotation summary of the assembled transcripts using various databases.

Database	Number of transcripts	% of total transcripts	Number of Unigenes	% of total Unigenes
Number of sequences	125,156	100	103,664	100
**Trinotate annotated**	**47**,**529**	**37**.**98**	**35**,**378**	**34**.**13**
SPROT_TOP_BLASTX_HIT	24,781	19.8	19,284	18.6
RNAMMER	20	0.02	17	0.02
SPROT_TOP_BLASTP_HIT	33,066	26.42	23,314	22.49
PFAM	30,846	24.65	21,458	20.7
SIGNALP	1,771	1.42	1,309	1.26
TMHMM	6,979	5.58	4,880	4.71
EGGNOG	35,195	28.12	26,088	25.17
KEGG	33,611	26.86	24,904	24.02
GENE_ONTOLOGY_BLAST	41,282	32.98	30,855	29.76
GENE_ONTOLOGY_PFAM	20,618	16.47	14,222	13.72
**Mercator annotated**	**39**,**860**	**31**.**84**	**28**,**236**	**27**.**23**
**Total annotated**	**59**,**699**	**47**.**70**	**41**,**698**	**40**.**22**

### Differentially expressed genes between the extreme segregating groups

We conducted differential gene expression analysis between the two extreme segregating groups. A total of 10,115 genes were identified as significantly differentially expressed genes (DEGs) in leaf tissue, while only 728 DEGs were detected in internode tissue between the two extreme groups. Among the 10,115 DEGs identified in leaf tissue, 5,495 displayed higher levels of expression in the high-biomass group and 4,620 showed higher levels of expression in the low-biomass group. In internode tissue, 304 DEGs expressed at higher levels in the high-biomass group and 424 expressed at higher levels in the low-biomass group. Detailed information of differential gene expression analysis is given in Supplemental Table S4.

We further assigned the DEGs to metabolic pathways using Mercator in order to identify major metabolic pathways controlling biomass accumulation in sugarcane. About 42% of the DEGs from leaf tissue and 52% of the DEGs from internode tissue could be assigned to the major functional bins using Mercator (Supplemental Table S5). Photosynthesis was the most highly overrepresented functional category in the DEGs whose expression was up-regulated in leaf of the high-biomass group. Other major enriched functional categories of the DEGs whose expression were up-regulated in leaves of the high-biomass group included tetrapyrrole synthesis, major carbohydrate (CHO) metabolism, and oxidative pentose phosphate pathway (OPP) (Supplemental Table S5). In the DEGs with up-regulated expression in leaves of the low-biomass group, fermentation and polyamine metabolism were the most overrepresented functional categories. Although leaf and internode tissues displayed different functional enrichment patterns, major carbohydrate metabolism, TCA, and cell wall precursor synthesis were the major enriched functional categories in both tissues of the high-biomass group. Interestingly, stress-related genes were highly enriched in the DEGs whose expression was up-regulated in internodes of the low-biomass group.

**Figure 2:**
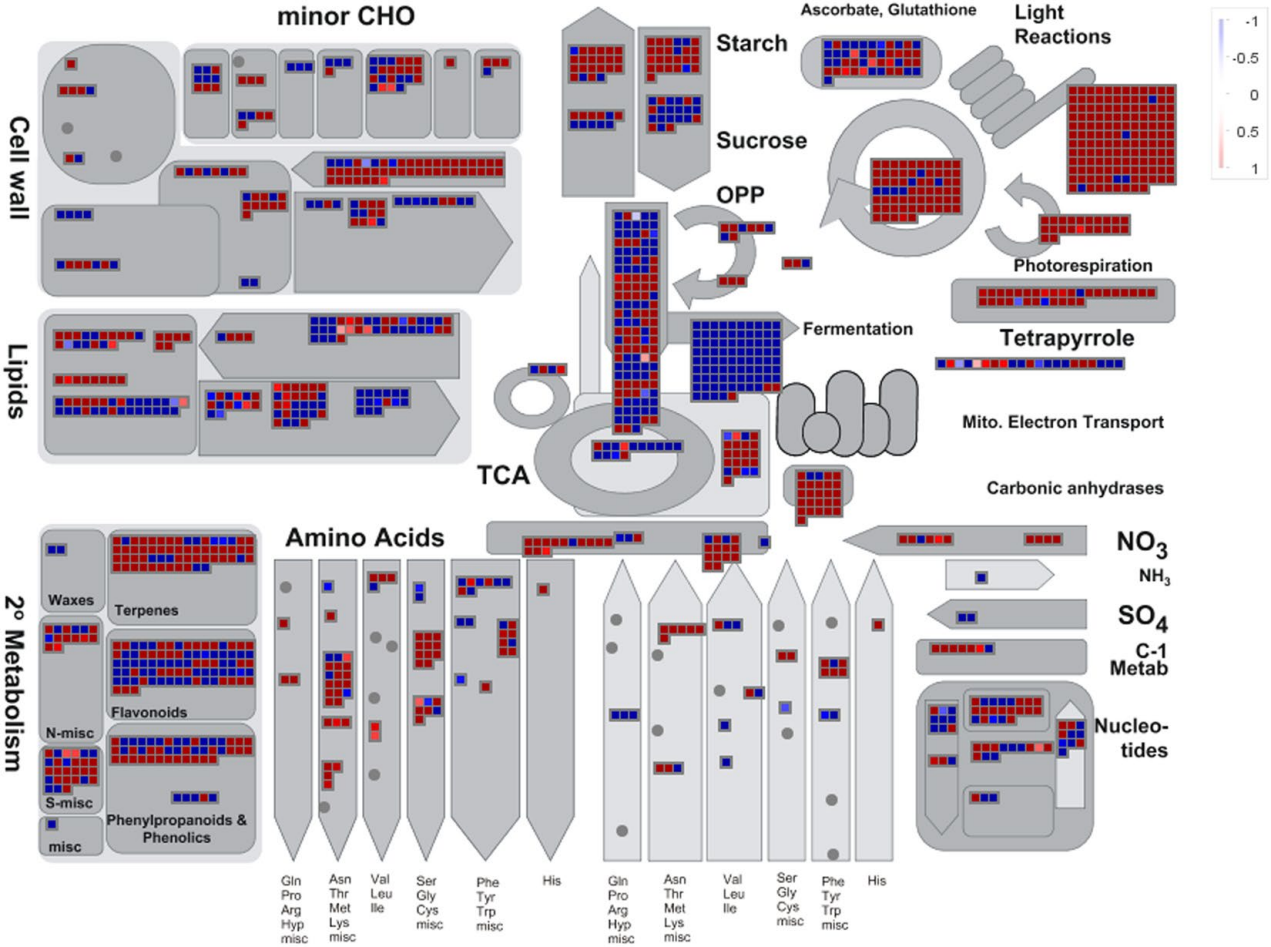
A metabolic overview of differentially expressed genes between the two extreme segregating groups. The expression levels of each gene are color coded in red-white-blue color scale, where red represents the highest expression, blue represents the lowest expression, and white represents an intermediate expression in the high-biomass group.

**Figure 3:**
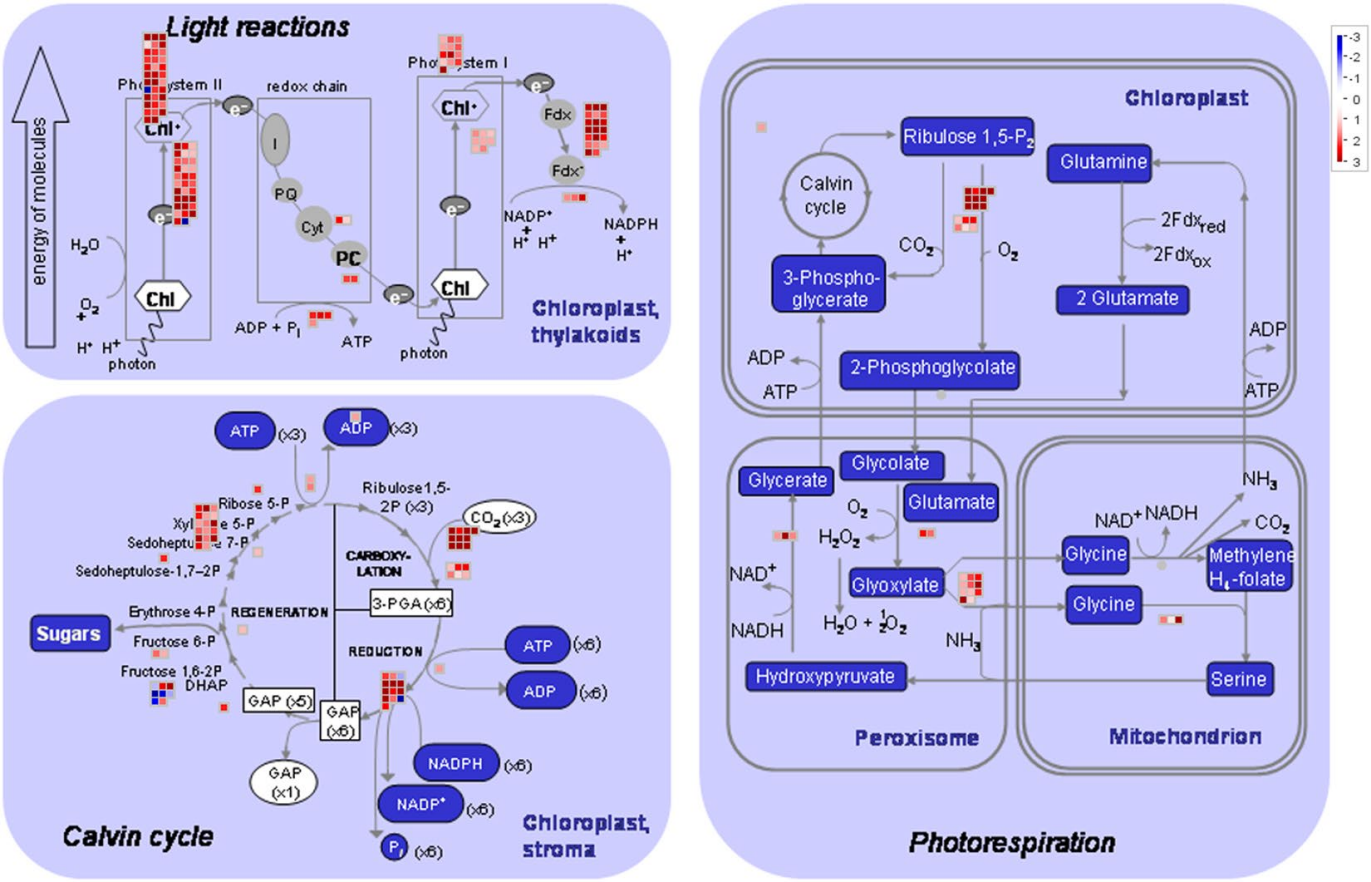
An overview of photosynthesis-related differentially expressed genes between the two extreme segregating groups. The expression levels of each gene are color coded in red-white-blue color scale, where red represents the highest expression, blue represents the lowest expression, and white represents an intermediate expression in the high-biomass group.

In leaves of the high-biomass group, the most enriched photosynthesis-associated genes were involved in the light reactions and the Calvin cycle (Fig. 2; Supplemental Fig. S2). In the functional category of light reactions, photosystem II and electron transport were the most significantly enriched (Fig. 3; Supplemental Fig. S2). Among the Calvin cycle related DEGs, genes coding for glyceraldehyde 3-phosphate (GAP), D-ribulose-5-phosphate 3-epimerase (RPE) and phosphoribulokinase (PRK) were the most enriched (Supplemental Fig. S3). Genes responsible for cell wall precursor synthesis were enriched in both leaf and internode tissues of the high-biomass group. Cellulose synthase genes and cellular cytoskeleton related genes were uniquely enriched in internodes of the high-biomass group. In addition, genes encoding major enzymes in lignin and starch biosynthesis pathways, such as cinnamyl alcohol dehydrogenase (CAD) and ADP glucose pyrophosphorylase (AGPase), were highly enriched in the high-biomass group.

In contrast to high-biomass group, DEGs with up-regulated expression levels in the low-biomass group were enriched in fermentation, hormone metabolism, stress, and signaling associated functional categories (Fig. 2, Supplemental Fig. S4). Alcohol dehydrogenase (ADH) and pyruvate decarboxylase (PDC), key enzymes in the fermentation process, were highly expressed in the low-biomass group (Supplemental Fig. S4). Genes involved in reactive oxygen species (ROS), an unavoidable consequence of aerobic metabolism, were enriched in the low-biomass group as well. Genes related to auxin, cytokinin, jasmonate, salicylic acid, and ethylene metabolism were also highly enriched in the low-biomass group. Among signaling related genes, legume-lectins, thaumatin-like, wheat LRK10-like, S-locus glycoprotein-like, wall associated kinase, and leucine rich repeats were the most enriched. Among stress associated genes, wounding and cold response genes in the abiotic sub-category and mildew resistance locus O (Mlo) in the biotic sub-category were highly enriched in the low-biomass group (Supplemental Fig. S5). In addition, genes associated to cell wall degradation processes, such as pectate lyase and polygalacturonase, were highly enriched in the low-biomass group.

### Functional classification of differentially expressed genes using gene ontology (GO) term enrichment analysis

We performed GO term enrichment analysis to further identify gene categories or pathways affecting biomass yield in sugarcane. In leaves of the high-biomass group, genes associated with chloroplast biogenesis, such as thylakoid membrane formation, proplastid development, and biosynthesis of photosynthetic pigments, were highly enriched biological process terms (Supplemental Table S6). Photosynthesis related biological process terms, such as light harvesting, electron transport chain, response to high light intensity, and regulation of photosynthesis, were highly enriched in leaves of the high-biomass group as well. Other highly enriched biological process terms in the high-biomass group included starch biosynthesis and metabolic process, carbohydrate biosynthetic and metabolic process, and cell wall precursor biosynthesis (Supplemental Table S6). In the cellular component ontology, the most enriched terms were chloroplast structure related components. In the molecular function ontology, UDP-glucose 4,6-dehydratase activity, malate dehydrogenase activity, ATP dependent peptidase activity, glucose-1-phosphate adenylyltransferase activity, coenzyme binding, cofactor binding, rRNA binding, and catalytic activity were the most enriched.

In internodes of the high-biomass group, the most enriched biological process terms included energy reserve metabolism, cellular glucan metabolism, carbohydrate metabolism, cellular amide metabolism. The most enriched cellular component term in internodes of the high-biomass group was amyloplast and the most enriched molecular function terms were glucose-1-phosphate adenylyltransferase activity, aldehyde dehydrogenase (NAD) activity, galactosidase activity, and magnesium ion binding.

In leaves of the low-biomass group, stress response, especially to oxidative stress, was the most highly enriched biological process term (Supplemental Table S7). GO terms related to oxidative stress response, such as hydrogen peroxide metabolism, reactive oxygen species (ROS) metabolism, and antioxidant activity, were highly enriched. Other highly enriched biological process terms included hormone-mediated signaling pathways, defense response, and regulation of flavonoid biosynthesis (Supplemental Table S7). In the cellular component ontology, plasma membrane and cell wall related genes were the most enriched. In the molecular function ontology, antioxidative activities, vitamin-related activities, and carbohydrate metabolism-related activities were highly enriched (Supplemental Table S7). Our GO term analysis results were consistent with the enriched biological processes identified by Mercator annotation of DEGs.

### Allele-specific expression in the extreme biomass groups

We used SNP markers to distinguish different alleles of each gene and then conducted allele-specific expression analysis. A total of 643 SNP loci from 423 genes were identified to show group-specific expression between the two extreme biomass groups (Supplemental Table S8). Among the 423 genes with group-specific expression alleles, 184 could be assigned functions by annotation. Detailed information of functional categories of the genes with group-specific expression alleles is given in Table 3. Seven alleles of 7 photosynthesis-related genes showed group-specific expression patterns. Five of them showed expression only in the high-biomass group while two of them showed expression only in the low-biomass group (Supplemental Table S8). All the 7 photosynthesis-related genes have functions in light reactions of photosynthesis. A total of 4 alleles of 4 fermentation-related genes were identified to be group-specific expression alleles (Table 3). Interestingly, all the 4 alleles were only expressed in the low-biomass group (Supplemental Table S8). These four fermentation-related genes encode key enzymes of the fermentation process, namely, an alcohol dehydrogenase (ADH) and three pyruvate decarboxylase (PDC). Alleles of 11 stress-related genes showed group-specific expression patterns. Seven of them were only expressed in the low-biomass group and four were only expressed in the high-biomass group (Supplemental Table S8).

**Table 3:** Functional categories of the genes with group-specific expression alleles.

Bin ID	Bin Description	High-biomass group	Low-biomass group
1	Photosynthesis	5	2
2	major CHO metabolism	1	4
3	minor CHO metabolism	1	1
4	glycolysis	6	1
5	fermentation	0	4
8	TCA/org transformation	5	1
9	mitochondrial electron transport/ATP synthesis	1	0
10	cell wall	1	1
11	lipid metabolism	3	2
13	amino acid metabolism	5	0
15	metal handling	3	0
16	secondary metabolism	3	4
17	hormone metabolism	2	7
18	Co-factor and vitamin metabolism	2	0
20	stress	4	7
21	redox	4	1
22	polyamine metabolism	0	1
23	nucleotide metabolism	2	1
26	Misc.	4	5
27	RNA	9	16
28	DNA	1	2
29	protein	16	21
30	signaling	4	8
33	development	2	2
34	transport	4	6
35	not assigned	125	117

## Discussion

As a C_4_ species, sugarcanes and energy canes are among the most efficient crops in converting solar energy into chemical energy. They are also among the leading crops with highly favorable input/output energy ratios^1^ and therefore, are prime candidates as biomass feedstocks. However, traditional sugarcane/energy cane breeding programs are time-consuming and expensive due to the large genome size, high ploidy level, complex genome structure and inheritance. Therefore, understanding the genetic and molecular basis of biomass yield in sugarcane/energy cane is important for future molecular breeding efforts to increase biomass yield in sugarcane/energy cane.

All modern sugarcane varieties are hybrids derived from interspecific hybridization between *Saccharum* species. Since *Saccharum* species possess 2n + n maternal chromosome transmission in certain crosses and backcrosses, modern sugarcane varieties have complicated genetics and very high aneuploidy chromosome numbers^11^. In addition, each allele may occur in 5–14 copies in the sugarcane genome^24^. Therefore, simple Mendelian inheritance rules do not apply to sugarcane in general due to complicated segregation statistics and interactions between alleles. In this study, we created a segregating population derived from an interspecific cross between *S*. *officinarum* and *S*. *spontaneum*, which can reflect the typical genomic structure and genetic background of modern sugarcane genomes. Transgressive segregation in biomass yield of the F2 individuals may be explained by a wide range of allele combinations caused by the high ploidy level and the large number of different alleles. Changes in allelic combination or copy number may subsequently alter the pattern and level of gene expression and result in the formation of extreme phenotypes. High yield potential of sugarcane/energy canes can be attributed to the presence of specific alleles, or different copy number of specific alleles, or a combination of different alleles. In our differential gene expression analysis, a total of 10,510 genes were identified to be significantly differentially expressed genes between the two extreme segregating groups, which accounts for 10% of the total assembled unigenes. Differential gene expression might be caused by allelic variations in regulatory regions or allelic interactions^25,26^, which subsequently lead to phenotypic changes. Besides differentially expressed genes, 423 genes exhibited group-specific expression patterns between the two extreme biomass groups, suggesting that the presence of specific alleles also contributed to the extreme biomass yields.

Photosynthesis is the ultimate source of biomass production, and yield is therefore related to net whole-plant photosynthetic carbon dioxide (CO_2_) assimilation over the growing season. However, yield is not only determined by CO_2_ assimilation capacity, but also by the way that assimilates are partitioned/utilized throughout the plant. Hence, biomass production is determined by the balance between carbon assimilation in source tissues (photosynthesis) and assimilate partitioning among sinks (for storage or metabolism). Low sink demand can lead to assimilate accumulation in source leaves and subsequently to decreased expression of genes coding for photosynthetic components, thus resulting in a reduced photosynthetic capacity. Therefore, sink capacity can regulate source activity^20–22^. For this reason, we sequenced transcriptomes of source tissues (top visible dewlap leaves) and sink tissues (9^th^ internode culm segments). Among the genes expressed in leaf tissue, 11.3% showed significantly differential expression patterns between the two extreme biomass groups. In contrast, less than 1% of genes that were expressed in internode tissue exhibited significant differential expression between the two extreme groups. Our result may suggest that source activities play a central role in achieving high biomass yield in sugarcane.

Biomass accumulation in plants with sufficient irrigation and mineral nutrition is mainly determined by solar radiation interception/absorption and the photosynthetic efficiency of light conversion into dry matter^19^. In our study, the segregating population were grown under identical, stress-free conditions with ample water and nutrient supply. Our differential gene expression analysis revealed that photosynthesis-related genes were highly enriched in up-regulated DEGs in the high-biomass group, which may explain the high photosynthetic efficiency in the high-biomass group. The genes associated with chloroplast biogenesis were highly enriched in the high-biomass group as well. Active proliferation of chloroplast in the high-biomass group may result in the high photosynthetic capacity and lead to high rates of light capture and a higher photosynthetic efficiency. We identified seven photosynthesis-related alleles that showed group-specific expression. Five of them were only expressed in the high-biomass group and two were only expressed in the low-biomass group. The presence of these alleles may affect photosynthetic efficiency and subsequently result in differential biomass yield.

Approximately 50% to 80% of photoassimilates are exported from source leaves to non-photosynthetic tissues (sinks)^27^ for storage or to support growth. Plants have evolved a fine-tuning regulatory system to coordinate carbon assimilation, storage, and growth^28^. Carbon availability affects plant growth, which can be reflected in expression of the biosynthesis- and growth-related genes. Enhanced rates of photosynthesis can lead to rapid growth. Rapid consumption of photosynthates in sinks can have a feed-forward effect on photosynthesis and can further stimulate carbon availability towards new structural growth. In the high-biomass group, genes responsible for cell wall precursor synthesis, lignin and starch biosynthesis, and cellular biosynthesis were highly enriched in the up-regulated DEGs. Active synthesis of structural components might result from high carbon availability. Rapid consumption of photosynthates could in turn help in maintaining high photosynthetic rates.

Fermentation was the most overrepresented functional category in leaves of the low-biomass group. Since the segregating population were grown under identical well irrigated/fertilized conditions, fermentative activity in the low-biomass group was not likely induced by external hypoxia. Plant internal oxygen concentrations are affected by energy-generating and -consuming metabolic activities. Zabalza *et al*. have shown that glycolytic activities regulate the availability of pyruvate for respiration and therefore affect the internal oxygen concentration^29^. Pyruvate kinase (PK), converting PEP directly into pyruvate, controls the production of pyruvate. Stimulation of glycolysis by pyruvate kinase (PK) has also been shown to lead to increased oxygen consumption^30^. Coincidently, our result showed that pyruvate kinase (PK) was expressed at a much higher level in the low-biomass group than in the high-biomass group. Furthermore, fermentation is not limited to anoxic conditions. Under aerobic conditions, fermentation plays an important role in balancing the level of pyruvate in the cell^29^. Enzymes that are involved in fermentative metabolism are induced primarily by a drop in the energy status of the tissue rather than by a low oxygen concentration^29^. Therefore, the high-level expression of fermentative genes in the low-biomass group was likely induced by their low-energy status. Compared to aerobic respiration, fermentation is inefficient in converting energy resources into ATP, which might further account for low-biomass yields.

## Conclusions

Transgressive segregation in the F2 population has resulted from a wide range of allele combinations due to the high ploidy level and a large number of different alleles. High-biomass yield was largely associated with carbon assimilation in source tissues than with sink tissue strength. The high-level expression of fermentative genes in the low-biomass group was likely induced by their low-energy status, which might also attribute to the low-biomass yield. A set of group-specific expression alleles were identified, which can be applied in the development of new high-yielding energy cane varieties via molecular breeding.

## Methods

### Development of the segregating population and field evaluation of biomass yield

An interspecific cross between *S*. *officinarum* LA Purple (2n = 80) × *S*. *spontaneum* US56-14-4 (2n = 80) was made at Hawaii Agriculture Research Center in 2010. A total of 120 F2 plants were generated and grown at the Kunia and Maunawili Stations, Oahu, Hawaii in 2012 and 2013. Since no experiment, such as chromosome counting or flow cytometry, was carried out to determine the chromosome numbers of the F1 and F2 individuals, it’s unclear whether the segregation population were derived from 2n + n chromosome transmission. Forty-seven F2 individuals were evaluated for field agronomic performance along with the parent LA Purple and the F1 10-9202 in 2015 and 2016. *S*. *spontaneu*m is listed as a Federal Noxious Weed by USDA-APHIS and is prohibited from field planting. Therefore, the parent US56-14-4 was not included in the field evaluation.

Seed pieces of the F2 individual were planted in 1.5 m × 1.5 m plots replicated three times and arranged in a Randomized Complete Block Design (RCBD). Stalk volume-related morphological data, including stalk diameter, stalk height, and stalk number, were measured 8.5 months after planting. Stalk diameter and stalk height were measured on three stalks per plot and the mean value was used to calculate stalk volume. Stalk volume was calculated using the formula:

1 $${\rm{V}}={\rm{\pi }}{\rm{\cdot }}{{\rm{r}}}^{2}{\rm{\cdot }}{\rm{h}}{\rm{\cdot }}{\rm{N}}$$

where r = mean radius of 3 stalks, h = mean height of 3 stalks, N = total number of stalk per plot. Dry weight was calculated using the formula:

$${\rm{dry\; weight}}={\rm{fresh\; weight}}{\rm{\times }}(1{\rm{-}}{\rm{moisture\; content}})$$

Five stalks per plot were harvested and shredded for moisture content measurement. Dry weight was calculated for each plot and averaged for 3 plots per clone. Dry weight data was collected from the parent LA Purple, the F1 10-9202, and 20 F2 clones 12 months after planting. ANOVA analysis for RCBD design was done using Genstat v17.

### Total RNA extraction and RNA-Seq library construction

The top visible dewlap leaf and the 9^th^ internode culm segment were harvested from each selected clone, flash frozen in liquid nitrogen, and stored in a freezer at −80 °C until RNA extraction. The tissues were ground to a fine powder in pre-cooled mortars. Total RNA was extracted using Isol-RNA Lysis Reagent (5 PRIME) following the manufacturer’s protocol. An additional isopropanol cleanup step was used to remove contaminants and improve the quality of the total RNA. The quality and integrity of the RNA samples were determined by running on an agarose gel and using a NanoDrop 2000 (Thermal Scientific). RNA-Seq libraries were constructed using KAPA Stranded mRNA-Seq Kit (Kapa Biosystems) following the manufacturer’s protocol. RNA-Seq libraries were quantified using a Qubit Fluorometer (Invitrogen), pooled, and paired-end sequenced on an Illumina HiSeq. 2500 (Illumina).

### Raw RNA-Seq data processing, assembly, and differential gene expression analysis

The paired-end raw reads were quality trimmed and overlapping pairs were merged before being assembled with Trinity^31^ using the following parameters –min_kmer_cov 2, –min_per_id_same_path 95, –max_diffs_same_path 8, –max_internal_gap_same_path 10, –kmer_size 31. Cleaned and merged reads from the parents LA Purple and US56-14-4 and the F1 10-9202 were combined and assembled with Trinity and used as reference assembly. RNA-Seq reads of the selected extreme segregants were mapped on the assembled reference transcriptome using bowtie 2^32^ and counted using RSEM^33^ to estimate the gene expression levels. Transcriptomes of the top visible dewlap leaf and the 9^th^ internode were analyzed separately for differential gene expression analysis. Differentially expressed genes were identified using the DESeq. 2 method. GO term enrichment analysis was performed using pipeline implemented in Trinity. The genes with ≥ 2-fold change and FDR corrected p-value < 0.05 were considered to be significantly differentially expressed. Significantly expressed genes were mapped onto bins using the Mercator web tool (http://mapman.gabipd.org/web/guest/app/mercator) and visualized using MapMan^34^.

### Transcriptome annotation

Assembled transcriptome was annotated using the Trinotate annotation suite v 3.0.1 (https://github.com/Trinotate/Trinotate). For annotation, TransDecoder^35^ was first used to predict the longest open reading frames (ORFs) in the transcripts. Transcripts and their translated protein sequences were then queried against the Trinotate version 3 specific releases of SwissProt and Pfam databases using BLASTX and BLASTP, respectively^36^. We then used the HMMER 3.1^37^ tool hmmscan and the Pfam-A database^38^ to annotate protein domains for each predicted protein sequence. Translated proteins were scanned for ribosomal RNAs, signal peptides and transmembrane topology using RNAmmer^39^, signalP^40^ and TMMHMM^41^, respectively. Transcripts were also searched through annotation databases eggnog, GO, and Kegg and the results were included in the final annotation of the transcripts. For high-resolution annotation, we used Mercator^42^, a web server tailored for plant omics data, to annotate assembled unigenes and differentially expressed genes. Mercator assigns each sequence to functional BINs that can be visualized on the pathways using MapMan^34^.

### Allele-specific expression analysis

Quality-trimmed reads were aligned to the assembled sugarcane transcriptome using bowtie 2^32^ with default alignment parameters. Bowtie 2 was instructed to add RG headers (LB, PL, PU, and SM) to the alignment files so that the alignment could be further used with freebayes. The resulting SAM files were sorted, converted to bam, indexed, and used as input for freebayes to call SNP with following parameter:–ploidy 12–use-best-n-alleles 4–pooled-continuous–min-coverage 3 -F 0.1–no-unal. Freebayes generates variant information in vcf format which was further processed with BCFtools^43^ to extract read count for each SNP. Read count data was TMM (trimmed mean of M values) normalized using edgeR^44^ with the help of run_TMM_scale_matrix.pl provided as support script with trinity package^31^. The normalized expression data was filtered to identify SNP which were uniquely present in all the members of one group but absent from other group in our comparison.

### Data Availability

The datasets generated during the current study are available in the NCBI SRA database accession numbers SRR4014615- SRR4014668 under BioProject PRJNA335885 (http://www.ncbi.nlm.nih.gov/bioproject/335885).

## Electronic supplementary material

Supplementary Figures S1-S5 Tables S1-S7

Supplementary Table S8
