# Supplementary material for: Transcriptomic analysis of transgressive segregants revealed the central role of photosynthetic capacity and efficiency in biomass accumulation in sugarcane
Source: Sci Rep. 2018 Mar 13;8:4415. doi: 10.1038/s41598-018-22798-5 (PMC5849761; doi:10.1038/s41598-018-22798-5)
Supplement: Supplementary file 1 — Supplementary Figures S1-S5 Tables S1-S7 [file 41598_2018_22798_MOESM1_ESM.pdf]

Supplementary information (Supplementary figures and tables)

Transcriptomic analysis of transgressive segregants revealed the central role of photosynthetic capacity and efficiency in biomass accumulation in sugarcane

Ratnesh Singh[1], Tyler Jones[2], Ching Man Wai[3], John Jifon[4], Chifumi Nagai[2], Ray Ming[3,5], Qingyi Yu [1,5,6*]

Author Affiliations

[1]Texas A&M AgriLife Research Center at Dallas, Texas A&M University System, Dallas, TX 75252, USA

[2] Hawaii Agriculture Research Center, Kunia, HI 96759, USA

[3]Department of Plant Biology, University of Illinois at Urbana-Champaign, Urbana, IL 61801, USA

[4]Texas A&M AgriLife Research Center at Weslaco, Texas A&M University System, Weslaco, TX 78596, USA

[5]Center for Genomics and Biotechnology, Fujian Provincial Key laboratory of Haixia applied plant systems biology, Haixia Institute of Science and Technology, Fujian Agriculture and Forestry University, Fuzhou, Fujian Province, China

[6] Department of Plant Pathology & Microbiology, Texas A&M University, College Station, TX 77843, USA

[*] To whom correspondence should be addressed: Qingyi Yu, Tel. +1 972-952-9225, Fax +1-972-952-9216, E-mail: qyu@ag.tamu.edu

Table of contents:

Supplementary Figure S1. Pie chart summary of assembled reference sequences annotated in major functional categories. The number of sequences in each category is shown following the functional categories.

Supplementary Figure S2. Expression profiling of photosynthesis-related differentially expressed genes in leaf of the high-biomass and low-biomass groups.

Supplementary Figure S3. An overview of differentially expressed genes in glycolysis-TCA cycle. The expression levels of each gene are color coded in red-white-blue color scale, where red represents the highest expression, blue represents the lowest expression, and white represents an intermediate expression in the high-biomass group.

Supplementary Figure S4. Expression profiling of fermentation-related differentially expressed genes in leaf of the high-biomass and low-biomass groups.

Supplementary Figure S5. An overview of cell response-related differentially expressed genes. The expression levels of each gene are color coded in red-white-blue color scale, where red represents the highest expression, blue represents the lowest expression, and white represents an intermediate expression in the high-biomass group.

Supplementary Table S1. Estimated stalk volume of the 47 F2 individuals along with the parent LA Purple (*S. officinarum*) and the F1 10-9202.

Supplementary Table S2. Statistical summary of the *de novo* transcriptome assembly. The RNA-Seq reads from the two parents, LA-Purple and US56-14-4, and the F1 10-9202 were combined to assemble the reference transcriptome.

Supplementary Table S3. Comparison of sugarcane assembled sequences to *Sorghum bicolor* CDSs.

Supplementary Table S4. Summary of differentially expressed genes in leaf and internode tissues between the high-biomass and low-biomass groups.

Supplementary Table S5. Fisher's exact test of enrichment for differently expressed genes in leaves and internodes between the low- and high-biomass groups. Cells with p-values < 0.001 are highlighted in red and p-value<=0.01 in green.

Supplementary Table S6. List of highly enriched GO terms of DEGs whose expression was up-regulated in the leaf of the high-biomass group.

Supplementary Table S7. List of highly enriched GO terms of DEGs whose expression was up-regulated in the leaf of the low-biomass group.

Supplementary Table S8. List of genes that showed allele-specific expression patterns between the two extreme biomass groups. Supplementary Table S8 is presented as a separate file in supplementary dataset.

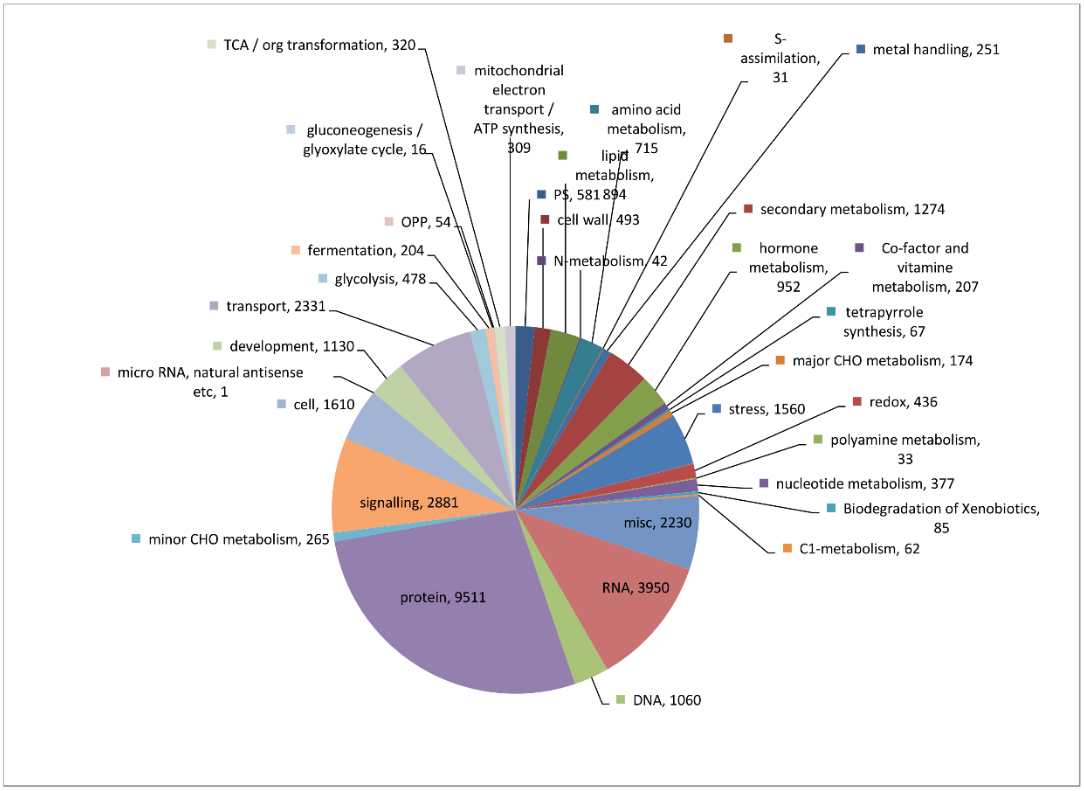

Supplementary Figure S1. Pie chart summary of assembled reference sequences annotated in major functional categories. The number of sequences in each category is shown following the functional categories.

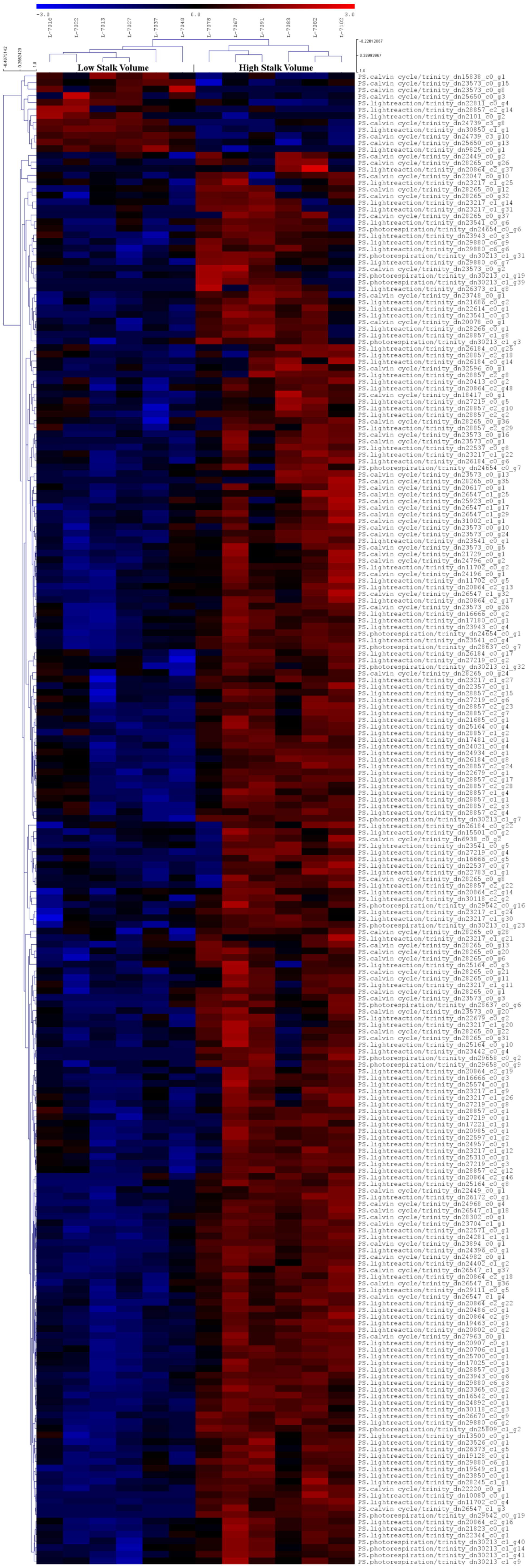

Supplementary Figure S2. Expression profiling of photosynthesis-related differentially expressed genes in leaf of the high-biomass and low-biomass groups.

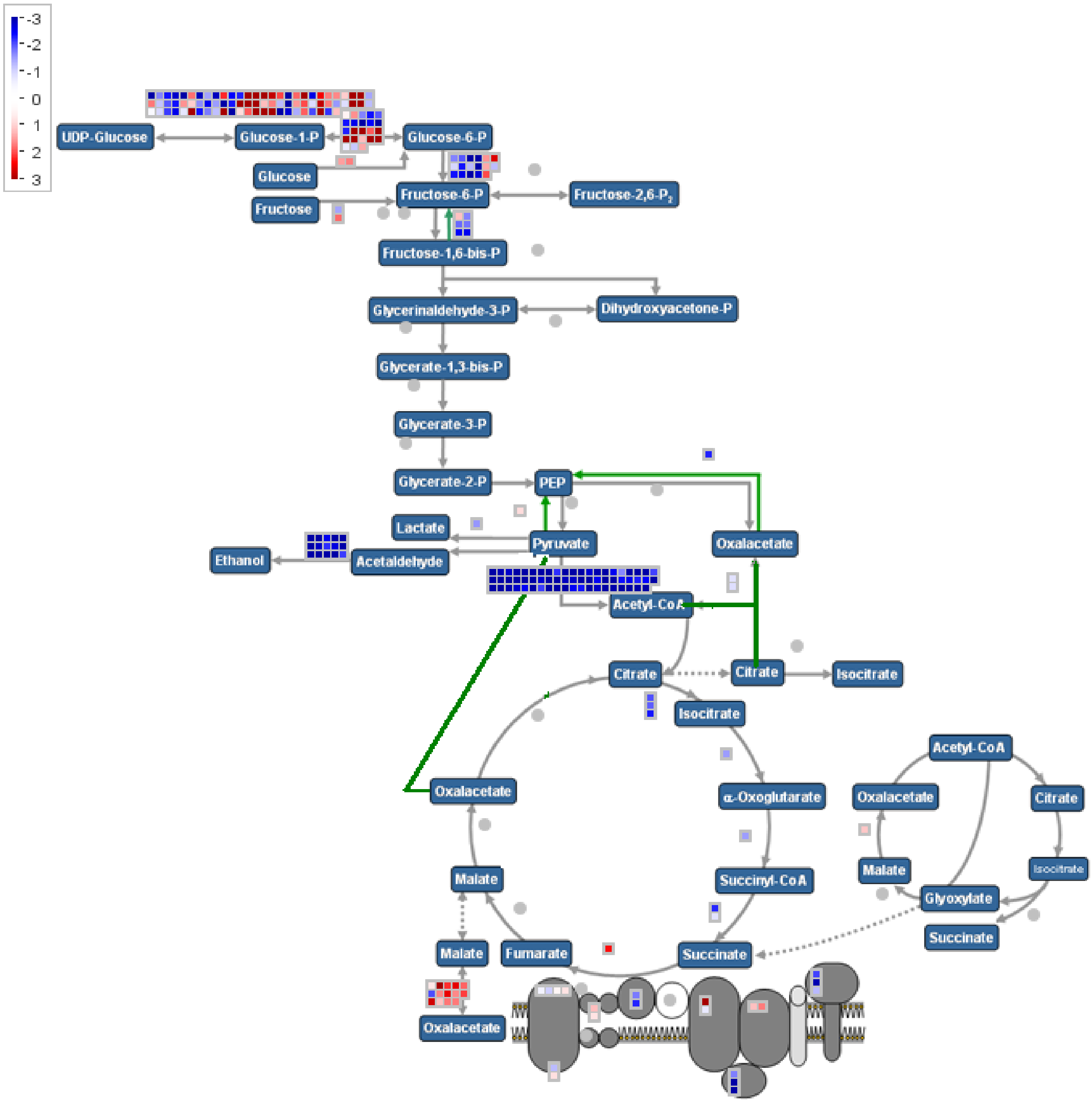

Supplementary Figure S3. An overview of differentially expressed genes in glycolysis-TCA cycle. The expression levels of each gene are color coded in red-white-blue color scale, where red represents the highest expression, blue represents the lowest expression, and white represents an intermediate expression in the high-biomass group.

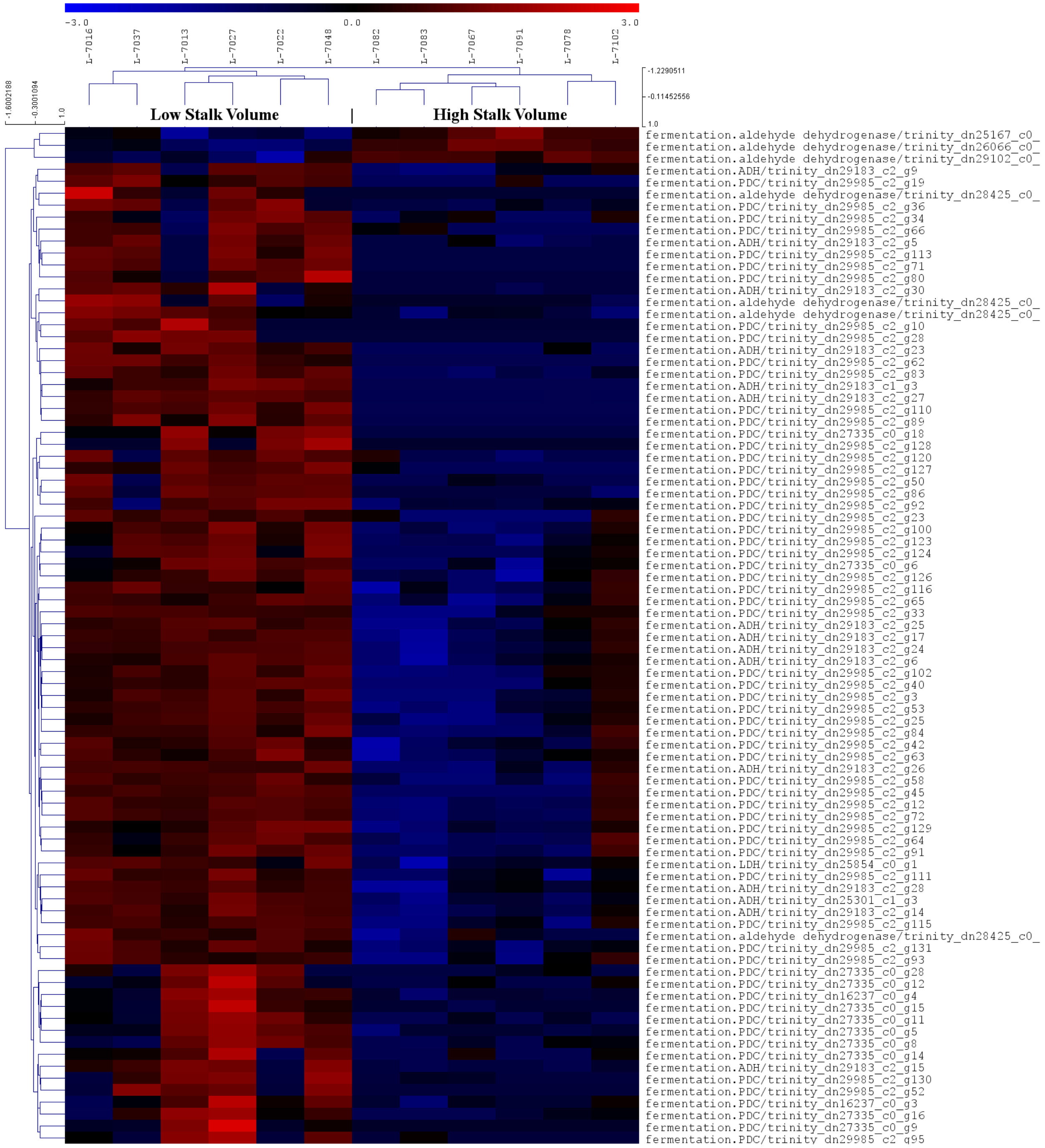

Supplementary Figure S4. Expression profiling of fermentation-related differentially expressed genes in leaf of the high-biomass and low-biomass groups.

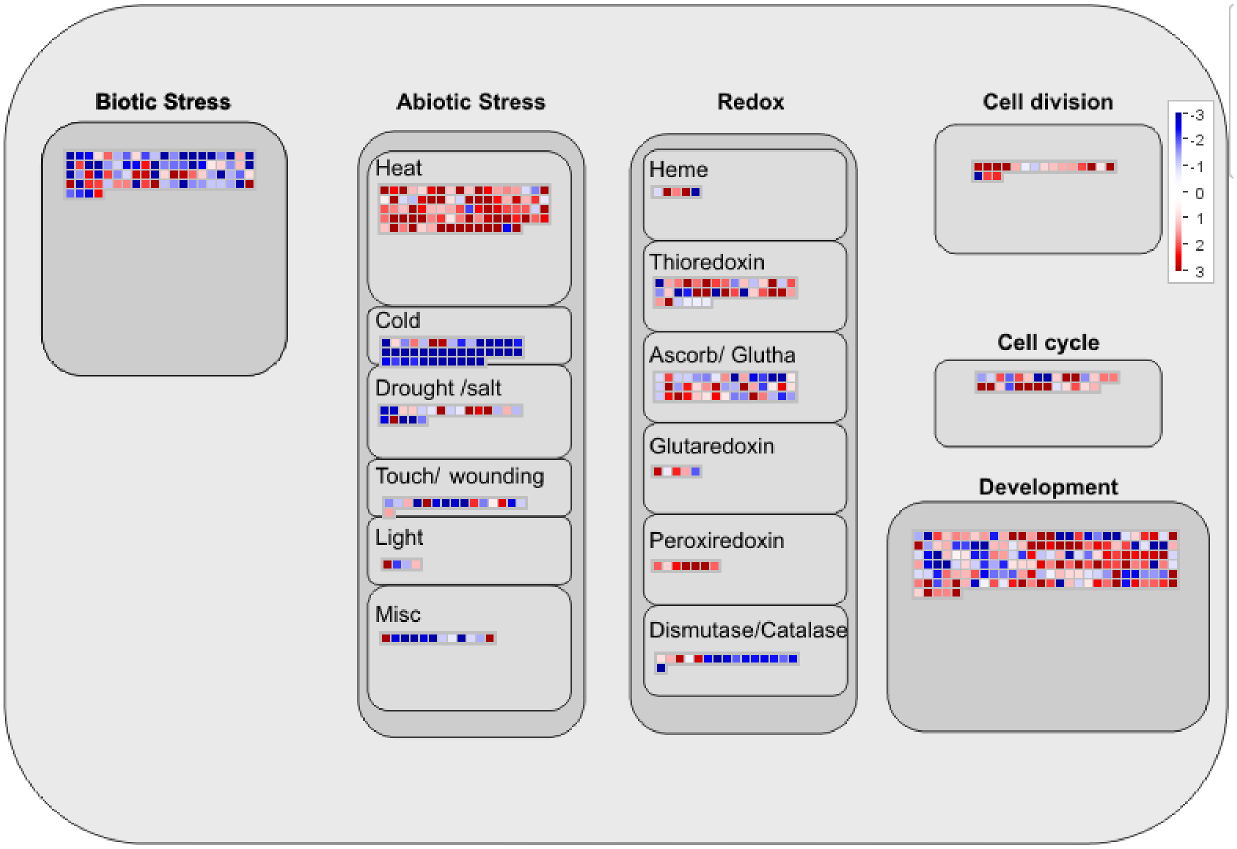

Supplementary Figure S5. An overview of cell response-related differentially expressed genes. The expression levels of each gene are color coded in red-white-blue color scale, where red represents the highest expression, blue represents the lowest expression, and white represents an intermediate expression in the high-biomass group.

Supplementary Table S1. Estimated stalk volume of the 47 F2 individuals along with the parent LA Purple (*S. officinarum*) and the F1 10-9202.

Rank	Clone ID	Stalk Volume (cm^3)	Dry Weight (Kg)	Group		Rank	Clone ID	Stalk Volume (cm^3)	Dry Weight (Kg)	Group
1	13-7078	67493	44	F2		26	12-7001	24423	19	F2
2	13-7082	61118	47	F2		27	12-7044	24386	19	F2
3	13-7083	60967	44	F2		28	13-7099	23404	-	F2
4	13-7102	49427	26	F2		29	12-7056	22874	-	F2
5	13-7067	46005	37	F2		30	13-7093	20711	-	F2
6	13-7091	45772	37	F2		31	12-7057	19117	-	F2
**7**	**10-9202**	**43737**	**36**	**F1**		32	13-7097	18469	-	F2
8	13-7094	38660	33	F2		33	12-7053	18139	-	F2
9	13-7098	37759	29	F2		34	12-7054	17972	-	F2
10	12-7051	36228	30	F2		35	12-7049	17814	-	F2
11	13-7074	35942	21	F2		36	12-7039	16766	-	F2
12	13-7064	34794	35	F2		37	12-7034	16620	-	F2
13	13-7090	34702	32	F2		38	12-7011	14153	-	F2
14	14-7128	34203	17	F2		39	12-7038	10879	-	F2
15	13-7063	32079	28	F2		40	12-7036	9702	-	F2
16	13-7101	31024	26	F2		41	12-7005	9399	-	F2
17	13-7087	28965	32	F2		42	14-7120	8846	-	F2
18	13-7092	28810	-	F2		**43**	**LA-Purple**	**8787**	**4**	**Parent**
19	13-7084	28174	-	F2		44	12-7016	8623	-	F2
20	14-7121	27242	-	F2		45	12-7048	7120	-	F2
21	12-7006	27051	12	F2		46	12-7013	5822	-	F2
22	13-7079	26856	-	F2		47	12-7037	5438	-	F2
23	13-7069	25581	-	F2		48	12-7022	5121	-	F2
24	13-7103	25303	-	F2		49	12-7027	2306	-	F2
25	12-7003	24461	21	F2						

Supplementary Table S2. Statistical summary of the *de novo* transcriptome assembly. The RNA-Seq reads from the two parents, LA-Purple and US56-14-4, and the F1 10-9202 were combined to assemble the reference transcriptome.

	Total number	Total length (bp)	N50 (bp)	Median contig length (bp)	Mean contig length (bp)
Transcripts	125,156	77,221,432	893	367	617
Unigenes	103,664	54,507,395	621	329	525

Supplementary Table 3. Comparison of sugarcane assembled sequences to *Sorghum bicolor* CDSs.

Query	Subject	Number of CDSs in Query	Number of CDSs in subject	Number of Query Hits	Number of subject hits	Hits% in Query	Hits% in subject	Average Length of query	N50 of query	N25 of query	Number Sequences > 1Kb in query
Sugarcane CDS	*S. bicolor* CDS	103,664	33,032	65,632	20,286	63.31	61.41	526	621	1,546	11,260
Sugarcane annotated CDS	*S. bicolor* CDS	41,698	33,032	37,018	18,604	88.78	56.32	816	1,218	2,053	10,646
Sugarcane unannotated CDS	*S. bicolor* CDS	61,966	33,032	28,615	11,273	46.18	34.13	331	319	466	614
Sugarcane unannotated CDS	NCBI NR	61,966	-	44,348	-	71.57	-	331	319	466	614
S. bicolor CDS	-	33,032	-	-	-	-	-	1,178	1,549	2,341	16,562

Supplementary Table S4. Summary of differentially expressed genes in leaf and internode tissues between the high-biomass and low-biomass groups.

Tissue	p-value < 0.05				FDR Adjusted p-value < 0.05			
	DE > 2x	UP in high-biomass group	UP in low-biomass group	Shared by leaf and internode	DE > 2x	UP in high-biomass group	UP in low-biomass group	Shared by leaf and internode
Leaf	17,300	9,304	7,996	2,180	10,115	5,495	4,620	333
Internode	5,090	1,956	3,134		728	304	424	

Supplementary Table S5. Fisher's exact test of enrichment for differently expressed genes in leaves and internodes between the low- and high-biomass groups. Cells with p-values < 0.001 are highlighted in red and p-value<=0.01 in green.

Bin ID	Bin Description	Reference transcriptome	Up in leaf of high-biomass group		Up in leaf of low-biomass group		Up in internode of high-biomass group		Up in internode of low-biomass group	
		Number	Number	p-value	Number	p-value	Number	p-value	Number	p-value
1	Photosynthesis	581	214	2.30E-117	12	1	2	0.53	4	0.23
2	major CHO metabolism	174	50	6.50E-23	25	5.00E-07	12	3.40E-13	0	1
3	minor CHO metabolism	265	26	2.70E-03	24	1.40E-03	3	4.70E-02	0	1
4	glycolysis	478	56	6.40E-08	58	3.00E-11	4	0.059	0	1
5	fermentation	204	3	1	82	1.50E-54	3	2.50E-02	1	0.58
6	gluconeogenesis / glyoxylate cycle	16	1	0.59	1	0.53	1	4.70E-02	0	1
7	OPP	54	10	5.70E-04	4	0.24	2	1.20E-02	0	1
8	TCA / org transformation	320	41	3.40E-07	13	0.72	7	6.20E-05	1	0.74
9	mitochondrial electron transport / ATP synthesis	309	5	1	10	0.91	1	0.61	1	0.73
10	cell wall	493	73	9.00E-15	33	2.20E-02	13	5.40E-09	0	1
11	lipid metabolism	894	64	1.50E-02	74	1.30E-06	5	0.14	4	0.51
12	N-metabolism	42	8	1.60E-03	2	0.58	3	2.90E-04	0	1
13	amino acid metabolism	715	88	9.70E-13	30	0.73	13	3.90E-07	4	0.35
14	S-assimilation	31	0	1	2	0.42	0	1	0	1
15	metal handling	251	20	0.055	6	0.98	2	0.18	3	0.089
16	secondary metabolism	1274	135	1.40E-13	89	8.70E-05	4	0.54	6	0.44
17	hormone metabolism	952	57	0.23	124	5.50E-25	4	0.33	10	7.50E-03
18	Co-factor and vitamin metabolism	207	31	3.00E-07	13	0.16	2	0.13	0	1
19	tetrapyrrole synthesis	67	25	3.70E-15	1	0.96	0	1	0	1
20	stress	1560	121	5.80E-05	145	2.30E-15	13	1.10E-03	21	3.90E-06
21	redox	436	56	2.50E-09	46	2.20E-07	1	0.73	3	0.28
22	polyamine metabolism	33	2	0.54	8	1.00E-04	2	4.50E-03	0	1
23	nucleotide metabolism	377	32	8.50E-03	25	4.60E-02	6	1.10E-03	5	2.20E-02

24	Biodegradation of Xenobiotics	85	15	4.80E-05	3	0.76	0	1	2	5.00E-02
25	C1-metabolism	62	4	0.43	1	0.95	1	0.17	0	1
26	Misc.	2230	206	9.30E-14	225	9.60E-28	13	2.00E-02	17	1.40E-02
27	RNA	3950	268	9.50E-05	266	6.30E-10	16	0.15	25	2.80E-02
28	DNA	1060	48	0.91	49	0.51	0	1	3	0.82
29	protein	9511	681	2.00E-14	397	0.99	58	2.50E-07	35	0.81
30	signaling	2881	160	0.38	269	1.10E-27	7	0.77	31	2.30E-06
31	cell	1610	84	0.65	61	0.95	16	4.20E-05	2	0.99
32	micro RNA, natural antisense etc.	1	0	1	0	1	0	1	0	1
33	development	1130	95	1.90E-05	52	0.53	1	0.97	7	0.2
34	transport	2331	165	3.20E-04	187	2.90E-13	11	0.1	30	8.70E-08
35	not assigned	75402	3106	1	2734	1	107	1	245	1
	**Total Genes**	**103664**	**5495**		**4620**		**304**		**424**	

Supplementary Table S6. List of highly enriched GO terms of DEGs whose expression was up-regulated in the leaf of the high-biomass group.

Ontology	Category	Over represented p-value	Over represented FDR	GO term
CC	GO:0044435	3.47E-139	3.50E-135	plastid part
CC	GO:0044434	6.43E-139	3.50E-135	chloroplast part
CC	GO:0009536	3.98E-129	1.45E-125	plastid
CC	GO:0009507	1.48E-127	4.03E-124	chloroplast
CC	GO:0044436	3.13E-95	6.81E-92	thylakoid part
CC	GO:0009535	3.34E-85	5.20E-82	chloroplast thylakoid membrane
CC	GO:0055035	3.34E-85	5.20E-82	plastid thylakoid membrane
CC	GO:0034357	8.31E-85	1.01E-81	photosynthetic membrane
CC	GO:0042651	8.31E-85	1.01E-81	thylakoid membrane
CC	GO:0009570	5.11E-55	5.57E-52	chloroplast stroma
CC	GO:0009532	5.65E-55	5.60E-52	plastid stroma
CC	GO:0009579	3.90E-49	3.54E-46	thylakoid
CC	GO:0044444	7.12E-46	5.97E-43	cytoplasmic part
CC	GO:0009941	1.52E-38	1.18E-35	chloroplast envelope
CC	GO:0009526	3.90E-38	2.83E-35	plastid envelope
BP	GO:0015979	4.13E-30	2.81E-27	photosynthesis
CC	GO:0009521	1.74E-29	1.12E-26	photosystem
CC	GO:0031967	1.49E-28	8.52E-26	organelle envelope
CC	GO:0031975	1.49E-28	8.52E-26	envelope
CC	GO:0009534	1.97E-28	1.02E-25	chloroplast thylakoid
CC	GO:0031976	1.97E-28	1.02E-25	plastid thylakoid
CC	GO:0031984	3.75E-26	1.86E-23	organelle subcompartment
CC	GO:0009523	6.24E-25	2.95E-22	photosystem II
CC	GO:0010287	4.85E-21	2.20E-18	plastoglobule
BP	GO:0006091	2.08E-20	9.08E-18	generation of precursor metabolites and energy
CC	GO:0031969	4.40E-20	1.85E-17	chloroplast membrane
CC	GO:0042170	1.51E-19	6.11E-17	plastid membrane
BP	GO:0044711	1.01E-18	3.94E-16	single-organism biosynthetic process
CC	GO:0031977	1.40E-18	5.28E-16	thylakoid lumen
BP	GO:0005982	2.22E-18	7.95E-16	starch metabolic process
BP	GO:0019253	2.33E-18	7.95E-16	reductive pentose-phosphate cycle
BP	GO:0019685	2.33E-18	7.95E-16	photosynthesis, dark reaction
BP	GO:0010109	3.11E-18	1.03E-15	regulation of photosynthesis
BP	GO:0042548	9.89E-18	3.17E-15	regulation of photosynthesis, light reaction
MF	GO:0016491	2.17E-17	6.77E-15	oxidoreductase activity

BP	GO:0043467	1.83E-16	5.53E-14	regulation of generation of precursor metabolites and energy
BP	GO:0044710	6.72E-16	1.98E-13	single-organism metabolic process
CC	GO:0009522	2.30E-15	6.58E-13	photosystem I
BP	GO:0055114	5.25E-15	1.47E-12	oxidation-reduction process
BP	GO:0009657	6.49E-15	1.77E-12	plastid organization
BP	GO:0016051	1.16E-14	3.07E-12	carbohydrate biosynthetic process
CC	GO:0009654	1.38E-14	3.58E-12	photosystem II oxygen evolving complex
BP	GO:0009644	1.43E-14	3.62E-12	response to high light intensity
CC	GO:0009543	2.30E-14	5.58E-12	chloroplast thylakoid lumen
CC	GO:0031978	2.30E-14	5.58E-12	plastid thylakoid lumen
BP	GO:0022900	6.23E-14	1.48E-11	electron transport chain
BP	GO:0010304	6.99E-14	1.62E-11	PSII associated light-harvesting complex II catabolic process
BP	GO:0009765	1.11E-13	2.51E-11	photosynthesis, light harvesting
BP	GO:0009658	2.35E-13	5.22E-11	chloroplast organization
MF	GO:0016168	3.28E-13	7.15E-11	chlorophyll binding
BP	GO:0009642	3.41E-13	7.29E-11	response to light intensity
BP	GO:0010207	3.58E-13	7.51E-11	photosystem II assembly
BP	GO:0019252	4.72E-13	9.70E-11	starch biosynthetic process
CC	GO:0043231	5.93E-13	1.20E-10	intracellular membrane-bounded organelle
BP	GO:0006073	9.65E-13	1.91E-10	cellular glucan metabolic process
CC	GO:0043227	1.31E-12	2.53E-10	membrane-bounded organelle
BP	GO:0044042	1.32E-12	2.53E-10	glucan metabolic process
CC	GO:0044424	1.45E-12	2.73E-10	intracellular part
MF	GO:0004176	1.58E-12	2.92E-10	ATP-dependent peptidase activity
BP	GO:0008152	1.80E-12	3.23E-10	metabolic process
BP	GO:0009058	1.81E-12	3.23E-10	biosynthetic process
BP	GO:0010205	2.88E-12	4.98E-10	photoinhibition
BP	GO:0043155	2.88E-12	4.98E-10	negative regulation of photosynthesis, light reaction
BP	GO:0042440	3.32E-12	5.65E-10	pigment metabolic process
BP	GO:0009668	5.24E-12	8.65E-10	plastid membrane organization
BP	GO:0010027	5.24E-12	8.65E-10	thylakoid membrane organization
BP	GO:0044257	6.96E-12	1.13E-09	cellular protein catabolic process
BP	GO:0009767	1.07E-11	1.71E-09	photosynthetic electron transport chain
CC	GO:0009501	1.28E-11	2.02E-09	amyloplast
BP	GO:1901576	1.42E-11	2.21E-09	organic substance biosynthetic process
BP	GO:0010206	2.33E-11	3.57E-09	photosystem II repair
BP	GO:0046148	3.15E-11	4.76E-09	pigment biosynthetic process

BP	GO:0009110	3.19E-11	4.76E-09	vitamin biosynthetic process
MF	GO:0010280	7.36E-11	1.07E-08	UDP-L-rhamnose synthase activity
MF	GO:0050377	7.36E-11	1.07E-08	UDP-glucose 4,6-dehydratase activity
MF	GO:0004222	1.53E-10	2.19E-08	metalloendopeptidase activity
MF	GO:0016853	1.88E-10	2.66E-08	isomerase activity
BP	GO:0010253	2.12E-10	2.93E-08	UDP-rhamnose biosynthetic process
BP	GO:0033478	2.12E-10	2.93E-08	UDP-rhamnose metabolic process
MF	GO:0048037	2.26E-10	3.08E-08	cofactor binding
BP	GO:0044237	2.71E-10	3.65E-08	cellular metabolic process
BP	GO:0006766	3.12E-10	4.15E-08	vitamin metabolic process
BP	GO:0018298	7.10E-10	9.32E-08	protein-chromophore linkage
BP	GO:0015995	7.34E-10	9.52E-08	chlorophyll biosynthetic process
BP	GO:0030091	9.40E-10	1.20E-07	protein repair
CC	GO:0043229	1.06E-09	1.34E-07	intracellular organelle
BP	GO:0044281	1.14E-09	1.43E-07	small molecule metabolic process
BP	GO:0015994	1.51E-09	1.87E-07	chlorophyll metabolic process
CC	GO:0043226	1.58E-09	1.94E-07	organelle
BP	GO:0005975	1.63E-09	1.98E-07	carbohydrate metabolic process
BP	GO:0044264	2.04E-09	2.44E-07	cellular polysaccharide metabolic process
BP	GO:0044723	2.47E-09	2.90E-07	single-organism carbohydrate metabolic process
BP	GO:0044249	2.48E-09	2.90E-07	cellular biosynthetic process
MF	GO:0004462	4.71E-09	5.46E-07	lactoylglutathione lyase activity
BP	GO:0044262	6.48E-09	7.43E-07	cellular carbohydrate metabolic process
MF	GO:0008460	6.95E-09	7.89E-07	dTDP-glucose 4,6-dehydratase activity
BP	GO:0009773	7.04E-09	7.91E-07	photosynthetic electron transport in photosystem I
BP	GO:0006081	1.28E-08	1.42E-06	cellular aldehyde metabolic process
BP	GO:0019682	1.51E-08	1.66E-06	glyceraldehyde-3-phosphate metabolic process
BP	GO:0006779	1.57E-08	1.71E-06	porphyrin-containing compound biosynthetic process
BP	GO:0016556	1.87E-08	2.01E-06	mRNA modification
BP	GO:0006778	1.97E-08	2.10E-06	porphyrin-containing compound metabolic process
MF	GO:0016829	2.57E-08	2.72E-06	lyase activity
CC	GO:0009706	2.78E-08	2.91E-06	chloroplast inner membrane
CC	GO:0009528	3.63E-08	3.75E-06	plastid inner membrane
BP	GO:0033013	3.65E-08	3.75E-06	tetrapyrrole metabolic process
BP	GO:0051552	3.86E-08	3.82E-06	flavone metabolic process

BP	GO:0051553	3.86E-08	3.82E-06	flavone biosynthetic process
BP	GO:0051554	3.86E-08	3.82E-06	flavonol metabolic process
BP	GO:0051555	3.86E-08	3.82E-06	flavonol biosynthetic process
BP	GO:0031425	4.02E-08	3.95E-06	chloroplast RNA processing
BP	GO:0033014	5.21E-08	5.06E-06	tetrapyrrole biosynthetic process
BP	GO:0009052	6.41E-08	6.18E-06	pentose-phosphate shunt, non-oxidative branch
CC	GO:0044464	6.51E-08	6.23E-06	cell part
BP	GO:0009228	7.44E-08	6.99E-06	thiamine biosynthetic process
BP	GO:0042724	7.44E-08	6.99E-06	thiamine-containing compound biosynthetic process
BP	GO:0019323	8.58E-08	7.99E-06	pentose catabolic process
MF	GO:0004750	1.01E-07	9.32E-06	ribulose-phosphate 3-epimerase activity
BP	GO:0042364	1.15E-07	1.05E-05	water-soluble vitamin biosynthetic process
MF	GO:0051537	1.26E-07	1.14E-05	2 iron, 2 sulfur cluster binding
BP	GO:1901617	1.55E-07	1.39E-05	organic hydroxy compound biosynthetic process
BP	GO:0009768	1.68E-07	1.48E-05	photosynthesis, light harvesting in photosystem I
MF	GO:0031409	1.68E-07	1.48E-05	pigment binding
BP	GO:0009416	1.68E-07	1.48E-05	response to light stimulus
CC	GO:0010598	1.79E-07	1.56E-05	NAD(P)H dehydrogenase complex (plastoquinone)
BP	GO:0010218	2.12E-07	1.83E-05	response to far red light
BP	GO:0005977	2.20E-07	1.89E-05	glycogen metabolic process
BP	GO:0009624	2.39E-07	2.04E-05	response to nematode
MF	GO:0050662	2.43E-07	2.05E-05	coenzyme binding
MF	GO:0008237	2.62E-07	2.19E-05	metallopeptidase activity
CC	GO:0010319	3.61E-07	3.01E-05	stromule
BP	GO:0009902	4.21E-07	3.45E-05	chloroplast relocation
BP	GO:0051667	4.21E-07	3.45E-05	establishment of plastid localization
BP	GO:0016119	4.29E-07	3.46E-05	carotene metabolic process
BP	GO:0006112	4.29E-07	3.46E-05	energy reserve metabolic process
BP	GO:0010114	4.63E-07	3.71E-05	response to red light
BP	GO:0006767	6.05E-07	4.81E-05	water-soluble vitamin metabolic process
MF	GO:0016984	6.39E-07	5.04E-05	ribulose-bisphosphate carboxylase activity
BP	GO:0009250	7.03E-07	5.49E-05	glucan biosynthetic process
BP	GO:0016109	7.10E-07	5.49E-05	tetraterpenoid biosynthetic process
BP	GO:0016117	7.10E-07	5.49E-05	carotenoid biosynthetic process
BP	GO:0005978	9.14E-07	7.02E-05	glycogen biosynthetic process

MF	GO:0016836	9.81E-07	7.48E-05	hydro-lyase activity
BP	GO:0042793	1.11E-06	8.37E-05	transcription from plastid promoter
BP	GO:0006772	1.14E-06	8.51E-05	thiamine metabolic process
BP	GO:0042723	1.14E-06	8.51E-05	thiamine-containing compound metabolic process
BP	GO:0015976	1.42E-06	0.000105424	carbon utilization
MF	GO:0050661	1.64E-06	0.000119674	NADP binding
BP	GO:0005996	1.64E-06	0.000119674	monosaccharide metabolic process
BP	GO:0009314	1.91E-06	0.000138596	response to radiation
BP	GO:2000022	2.00E-06	0.000144092	regulation of jasmonic acid mediated signaling pathway
MF	GO:0046554	2.01E-06	0.000144092	malate dehydrogenase (NADP+) activity
BP	GO:0016108	2.05E-06	0.000145101	tetraterpenoid metabolic process
BP	GO:0016116	2.05E-06	0.000145101	carotenoid metabolic process
MF	GO:0016846	3.02E-06	0.000212127	carbon-sulfur lyase activity
MF	GO:0008686	3.04E-06	0.000212127	3,4-dihydroxy-2-butanone-4-phosphate synthase activity
BP	GO:0005976	4.92E-06	0.00034121	polysaccharide metabolic process
BP	GO:0019321	5.30E-06	0.000365681	pentose metabolic process
MF	GO:0003935	5.61E-06	0.000384779	GTP cyclohydrolase II activity
BP	GO:0071704	6.25E-06	0.00042548	organic substance metabolic process
MF	GO:0051536	7.62E-06	0.000512705	iron-sulfur cluster binding
MF	GO:0051540	7.62E-06	0.000512705	metal cluster binding
MF	GO:0003933	9.89E-06	0.000661142	GTP cyclohydrolase activity
BP	GO:0015977	1.04E-05	0.000689649	carbon fixation
BP	GO:0006536	1.08E-05	0.000712173	glutamate metabolic process
BP	GO:0035304	1.16E-05	0.000753149	regulation of protein dephosphorylation
BP	GO:0016143	1.17E-05	0.000753149	S-glycoside metabolic process
BP	GO:0019757	1.17E-05	0.000753149	glycosinolate metabolic process
BP	GO:0019760	1.17E-05	0.000753149	glucosinolate metabolic process
BP	GO:0010315	1.28E-05	0.000818942	auxin efflux
BP	GO:0051656	1.50E-05	0.000954055	establishment of organelle localization
BP	GO:0034637	1.51E-05	0.000954861	cellular carbohydrate biosynthetic process
BP	GO:0006457	1.66E-05	0.001043807	protein folding
MF	GO:0016835	1.67E-05	0.001043807	carbon-oxygen lyase activity
MF	GO:0004970	1.75E-05	0.001078717	ionotropic glutamate receptor activity
MF	GO:0005230	1.75E-05	0.001078717	extracellular ligand-gated ion channel activity
MF	GO:0008066	1.75E-05	0.001078717	glutamate receptor activity

MF	GO:0019843	1.82E-05	0.001111864	rRNA binding
MF	GO:0015276	1.91E-05	0.001159041	ligand-gated ion channel activity
MF	GO:0022834	1.91E-05	0.001159041	ligand-gated channel activity
MF	GO:0016887	2.08E-05	0.001250167	ATPase activity
BP	GO:0010155	2.19E-05	0.001309018	regulation of proton transport
BP	GO:0009231	2.31E-05	0.00137858	riboflavin biosynthetic process
BP	GO:0044802	2.42E-05	0.001435081	single-organism membrane organization
BP	GO:0009813	2.57E-05	0.001508135	flavonoid biosynthetic process
BP	GO:0046246	2.57E-05	0.001508135	terpene biosynthetic process
MF	GO:0008964	2.86E-05	0.001667392	phosphoenolpyruvate carboxylase activity
BP	GO:0016120	3.28E-05	0.001894402	carotene biosynthetic process
BP	GO:0006771	3.29E-05	0.001894402	riboflavin metabolic process
BP	GO:0042727	3.32E-05	0.001902336	flavin-containing compound biosynthetic process
BP	GO:0009628	3.42E-05	0.001949737	response to abiotic stimulus
BP	GO:0016144	3.50E-05	0.001964667	S-glycoside biosynthetic process
BP	GO:0019758	3.50E-05	0.001964667	glycosinolate biosynthetic process
BP	GO:0019761	3.50E-05	0.001964667	glucosinolate biosynthetic process
BP	GO:0009812	3.63E-05	0.002026704	flavonoid metabolic process
MF	GO:0003714	4.02E-05	0.002236034	transcription corepressor activity
BP	GO:0019722	4.43E-05	0.002442552	calcium-mediated signaling
BP	GO:0000023	4.46E-05	0.002442552	maltose metabolic process
BP	GO:0010360	4.57E-05	0.002442552	negative regulation of anion channel activity
BP	GO:0010361	4.57E-05	0.002442552	regulation of anion channel activity by blue light
BP	GO:0010362	4.57E-05	0.002442552	negative regulation of anion channel activity by blue light
BP	GO:0032410	4.57E-05	0.002442552	negative regulation of transporter activity
BP	GO:0032413	4.57E-05	0.002442552	negative regulation of ion transmembrane transporter activity
BP	GO:1903792	4.57E-05	0.002442552	negative regulation of anion transport
BP	GO:0006775	4.73E-05	0.002467753	fat-soluble vitamin metabolic process
BP	GO:0010189	4.73E-05	0.002467753	vitamin E biosynthetic process
BP	GO:0042360	4.73E-05	0.002467753	vitamin E metabolic process
BP	GO:0042362	4.73E-05	0.002467753	fat-soluble vitamin biosynthetic process
BP	GO:0005983	4.73E-05	0.002467753	starch catabolic process
MF	GO:0004089	5.08E-05	0.002626685	carbonate dehydratase activity
CC	GO:0019898	5.09E-05	0.002626685	extrinsic component of membrane

BP	GO:0046456	5.25E-05	0.002684367	icosanoid biosynthetic process
BP	GO:1901570	5.25E-05	0.002684367	fatty acid derivative biosynthetic process
BP	GO:0043436	6.11E-05	0.003110598	oxoacid metabolic process
MF	GO:0010181	6.18E-05	0.003122131	FMN binding
MF	GO:0004611	6.19E-05	0.003122131	phosphoenolpyruvate carboxykinase activity
BP	GO:0043200	6.38E-05	0.003193635	response to amino acid
BP	GO:0043623	6.39E-05	0.003193635	cellular protein complex assembly
BP	GO:0033692	6.72E-05	0.003342295	cellular polysaccharide biosynthetic process
CC	GO:0044446	6.79E-05	0.003364612	intracellular organelle part
BP	GO:0044283	6.88E-05	0.003393895	small molecule biosynthetic process
MF	GO:0015036	7.15E-05	0.003512078	disulfide oxidoreductase activity
BP	GO:0042726	7.53E-05	0.003679652	flavin-containing compound metabolic process
BP	GO:0071230	8.38E-05	0.004075496	cellular response to amino acid stimulus
CC	GO:0048046	8.60E-05	0.004166986	apoplast
BP	GO:0009637	8.70E-05	0.004191053	response to blue light
BP	GO:0006082	8.73E-05	0.004191053	organic acid metabolic process
BP	GO:1901564	9.41E-05	0.004499598	organonitrogen compound metabolic process
BP	GO:0009226	9.64E-05	0.004587657	nucleotide-sugar biosynthetic process
CC	GO:0044422	9.84E-05	0.004660709	organelle part
MF	GO:0009055	0.000101711	0.004798468	electron carrier activity
BP	GO:0010118	0.000102182	0.004799909	stomatal movement
BP	GO:0034763	0.000111204	0.005179079	negative regulation of transmembrane transport
BP	GO:0034766	0.000111204	0.005179079	negative regulation of ion transmembrane transport
MF	GO:0016667	0.000129975	0.006027504	oxidoreductase activity, acting on a sulfur group of donors
BP	GO:1901135	0.000133176	0.006149815	carbohydrate derivative metabolic process
BP	GO:0061024	0.000137735	0.006333463	membrane organization
MF	GO:0047100	0.000148047	0.006779044	glyceraldehyde-3-phosphate dehydrogenase (NADP+) (phosphorylating) activity
MF	GO:0016830	0.000162218	0.007396857	carbon-carbon lyase activity
BP	GO:0034599	0.000169939	0.007716626	cellular response to oxidative stress
BP	GO:0046365	0.000179976	0.008121009	monosaccharide catabolic process
BP	GO:0042214	0.000180334	0.008121009	terpene metabolic process
BP	GO:0042549	0.000192674	0.008640997	photosystem II stabilization

BP	GO:0010034	0.000197215	0.008757581	response to acetate
BP	GO:0071311	0.000197215	0.008757581	cellular response to acetate
MF	GO:0004351	0.000198082	0.008757581	glutamate decarboxylase activity
BP	GO:0005984	0.000198488	0.008757581	disaccharide metabolic process
BP	GO:0010323	0.000207395	0.009113663	negative regulation of isopentenyl diphosphate biosynthetic process, methylerythritol 4-phosphate pathway
BP	GO:0044724	0.000208541	0.00912722	single-organism carbohydrate catabolic process
BP	GO:0008299	0.000209737	0.009142874	isoprenoid biosynthetic process
MF	GO:0015035	0.000210651	0.009146125	protein disulfide oxidoreductase activity
MF	GO:0051920	0.000230633	0.009973975	peroxiredoxin activity
MF	GO:0008878	0.000244962	0.010551752	glucose-1-phosphate adenylyltransferase activity
BP	GO:0019932	0.000254385	0.010914527	second-messenger-mediated signaling
BP	GO:0051156	0.000260652	0.011139536	glucose 6-phosphate metabolic process
BP	GO:0006098	0.000287226	0.012227296	pentose-phosphate shunt
BP	GO:0009853	0.000290299	0.012310043	photorespiration
MF	GO:0004373	0.000298968	0.012628495	glycogen (starch) synthase activity
MF	GO:0016814	0.000311983	0.013127382	hydrolase activity, acting on carbon-nitrogen (but not peptide) bonds, in cyclic amidines
BP	GO:0016052	0.000315816	0.013237539	carbohydrate catabolic process
BP	GO:0010359	0.000322837	0.013479986	regulation of anion channel activity
MF	GO:0003824	0.000337307	0.014030411	catalytic activity
CC	GO:0000229	0.000363744	0.01501547	cytoplasmic chromosome
CC	GO:0009508	0.000363744	0.01501547	plastid chromosome
CC	GO:0009295	0.000367194	0.015100662	nucleoid
BP	GO:0000271	0.000404886	0.016588158	polysaccharide biosynthetic process
MF	GO:2001070	0.000435359	0.017769837	starch binding
MF	GO:0004654	0.000437129	0.017775486	polyribonucleotide nucleotidyltransferase activity
BP	GO:0071260	0.000448166	0.018156558	cellular response to mechanical stimulus
MF	GO:0019238	0.000464141	0.018734124	cyclohydrolase activity
BP	GO:0071072	0.000480318	0.019244501	negative regulation of phospholipid biosynthetic process
BP	GO:1903726	0.000480318	0.019244501	negative regulation of phospholipid metabolic process
BP	GO:0032544	0.000497618	0.019864637	plastid translation
BP	GO:0016036	0.00051224	0.02037368	cellular response to phosphate starvation

MF	GO:0004167	0.00051944	0.020510334	dopachrome isomerase activity
MF	GO:0050178	0.00051944	0.020510334	phenylpyruvate tautomerase activity
BP	GO:0009611	0.000552397	0.021732942	response to wounding
MF	GO:0016620	0.000567656	0.022252945	oxidoreductase activity, acting on the aldehyde or oxo group of donors, NAD or NADP as acceptor
MF	GO:0008171	0.000573418	0.022398233	O-methyltransferase activity
BP	GO:0009451	0.000590456	0.022981382	RNA modification
BP	GO:0009251	0.000602132	0.023291334	glucan catabolic process
MF	GO:0080045	0.000602694	0.023291334	quercetin 3'-O-glucosyltransferase activity
BP	GO:0010103	0.000634705	0.024441731	stomatal complex morphogenesis
BP	GO:0044763	0.000639152	0.024526326	single-organism cellular process
BP	GO:0008610	0.000653025	0.024887772	lipid biosynthetic process
MF	GO:0052923	0.000653138	0.024887772	all-trans-nonaprenyl-diphosphate synthase (geranyl-diphosphate specific) activity
MF	GO:1990137	0.000657557	0.024968824	plant seed peroxidase activity
BP	GO:0022898	0.000679828	0.025635874	regulation of transmembrane transporter activity
BP	GO:0032412	0.000679828	0.025635874	regulation of ion transmembrane transporter activity
CC	GO:0009368	0.000691958	0.025913945	endopeptidase Clp complex
CC	GO:0009840	0.000691958	0.025913945	chloroplastic endopeptidase Clp complex
MF	GO:0003844	0.000727837	0.027164288	1,4-alpha-glucan branching enzyme activity
MF	GO:0019203	0.000731238	0.027198046	carbohydrate phosphatase activity
MF	GO:0045551	0.00077104	0.028265538	cinnamyl-alcohol dehydrogenase activity
MF	GO:0052747	0.00077104	0.028265538	sinapyl alcohol dehydrogenase activity
BP	GO:0045036	0.000772906	0.028265538	protein targeting to chloroplast
BP	GO:0072596	0.000772906	0.028265538	establishment of protein localization to chloroplast
BP	GO:0072598	0.000772906	0.028265538	protein localization to chloroplast
BP	GO:0006720	0.00083427	0.030407605	isoprenoid metabolic process
MF	GO:0050347	0.000845452	0.030712447	trans-octaprenyltranstransferase activity
BP	GO:0016123	0.000908644	0.03289836	xanthophyll biosynthetic process
BP	GO:0032409	0.000915795	0.033047471	regulation of transporter activity
BP	GO:0006636	0.000930775	0.033380722	unsaturated fatty acid biosynthetic process
BP	GO:0045827	0.000931156	0.033380722	negative regulation of isoprenoid metabolic process

BP	GO:1901615	0.000948563	0.033886622	organic hydroxy compound metabolic process
MF	GO:0004740	0.000951487	0.033886622	pyruvate dehydrogenase (acetyl-transferring) kinase activity
BP	GO:0009225	0.000961659	0.034137314	nucleotide-sugar metabolic process
BP	GO:0071417	0.001010198	0.035743955	cellular response to organonitrogen compound
MF	GO:0016857	0.00101725	0.035876999	racemase and epimerase activity, acting on carbohydrates and derivatives
MF	GO:0009011	0.001051886	0.036859976	starch synthase activity
MF	GO:0033201	0.001051886	0.036859976	alpha-1,4-glucan synthase activity
BP	GO:0019693	0.001170386	0.039893505	ribose phosphate metabolic process
CC	GO:0009503	0.001171055	0.039893505	thylakoid light-harvesting complex
CC	GO:0009517	0.001171055	0.039893505	PSII associated light-harvesting complex II
CC	GO:0009783	0.001171055	0.039893505	photosystem II antenna complex
CC	GO:0030076	0.001171055	0.039893505	light-harvesting complex
MF	GO:0016719	0.00117417	0.039893505	carotene 7,8-desaturase activity
MF	GO:0052886	0.00117417	0.039893505	9,9'-dicis-carotene:quinone oxidoreductase activity
MF	GO:0052887	0.00117417	0.039893505	7,9,9'-tricis-neurosporene:quinone oxidoreductase activity
BP	GO:0052889	0.00117417	0.039893505	9,9'-di-cis-zeta-carotene desaturation to 7,9,7',9'-tetra-cis-lycopene
BP	GO:0010256	0.001175061	0.039893505	endomembrane system organization
CC	GO:0080085	0.001196082	0.040370472	signal recognition particle, chloroplast targeting
BP	GO:0006662	0.001200223	0.040370472	glycerol ether metabolic process
BP	GO:0018904	0.001200223	0.040370472	ether metabolic process
MF	GO:0000285	0.001216417	0.040789277	1-phosphatidylinositol-3-phosphate 5-kinase activity
BP	GO:0042180	0.001231109	0.041155299	cellular ketone metabolic process
MF	GO:0019172	0.001254155	0.041670049	glyoxalase III activity
BP	GO:0019249	0.001254155	0.041670049	lactate biosynthetic process
BP	GO:0019752	0.00126355	0.041854616	carboxylic acid metabolic process
BP	GO:1900864	0.001328073	0.043858599	mitochondrial RNA modification
MF	GO:0016859	0.001377911	0.045366996	cis-trans isomerase activity
BP	GO:0072527	0.001382207	0.045371349	pyrimidine-containing compound metabolic process
BP	GO:0033559	0.001388263	0.045433306	unsaturated fatty acid metabolic process
MF	GO:0030170	0.001496505	0.048829072	pyridoxal phosphate binding

Supplementary Table S7. List of highly enriched GO terms of DEGs whose expression was up-regulated in the leaf of the low-biomass group.

ontology	category	Over represented p-value	Over represented FDR	term
BP	GO:0042744	7.91E-46	8.62E-42	hydrogen peroxide catabolic process
BP	GO:0042743	5.05E-45	2.75E-41	hydrogen peroxide metabolic process
BP	GO:0072593	4.00E-40	1.45E-36	reactive oxygen species metabolic process
MF	GO:0004601	1.19E-39	3.23E-36	peroxidase activity
MF	GO:0016684	8.01E-36	1.75E-32	oxidoreductase activity, acting on peroxide as acceptor
MF	GO:0016209	1.28E-33	2.32E-30	antioxidant activity
MF	GO:0020037	1.39E-30	2.16E-27	heme binding
BP	GO:0044712	9.22E-28	1.26E-24	single-organism catabolic process
BP	GO:0006979	4.30E-27	5.21E-24	response to oxidative stress
MF	GO:0046906	6.67E-27	7.27E-24	tetrapyrrole binding
CC	GO:0005576	1.39E-26	1.38E-23	extracellular region
MF	GO:0004737	5.34E-24	4.85E-21	pyruvate decarboxylase activity
MF	GO:0019842	7.21E-24	6.04E-21	vitamin binding
BP	GO:0050896	1.60E-23	1.25E-20	response to stimulus
MF	GO:1901681	4.58E-22	3.32E-19	sulfur compound binding
MF	GO:0030976	1.76E-20	1.20E-17	thiamine pyrophosphate binding
MF	GO:0003700	1.26E-18	8.08E-16	transcription factor activity, sequence-specific DNA binding
MF	GO:0001071	1.50E-18	9.06E-16	nucleic acid binding transcription factor activity
BP	GO:0009056	6.53E-18	3.74E-15	catabolic process
BP	GO:0009755	2.55E-17	1.39E-14	hormone-mediated signaling pathway
MF	GO:0016491	1.04E-14	5.40E-12	oxidoreductase activity
BP	GO:0006950	6.30E-14	3.05E-11	response to stress
MF	GO:0016831	6.44E-14	3.05E-11	carboxy-lyase activity
BP	GO:0044248	3.22E-13	1.46E-10	cellular catabolic process
CC	GO:0005773	1.88E-11	8.20E-09	vacuole
BP	GO:0009873	3.20E-11	1.34E-08	ethylene-activated signaling pathway
MF	GO:0016830	6.51E-11	2.63E-08	carbon-carbon lyase activity
BP	GO:0042221	7.00E-11	2.72E-08	response to chemical
BP	GO:0000160	1.17E-10	4.39E-08	phosphorelay signal transduction system
BP	GO:0010033	2.41E-10	8.74E-08	response to organic substance
BP	GO:1901700	2.78E-10	9.79E-08	response to oxygen-containing compound
BP	GO:0044699	7.48E-10	2.55E-07	single-organism process
BP	GO:0055114	9.32E-10	3.08E-07	oxidation-reduction process
BP	GO:0044710	1.09E-09	3.50E-07	single-organism metabolic process
BP	GO:0007165	1.30E-09	4.05E-07	signal transduction

BP	GO:0001101	3.93E-09	1.19E-06	response to acid chemical
MF	GO:0004014	8.28E-09	2.31E-06	adenosylmethionine decarboxylase activity
BP	GO:0006557	8.28E-09	2.31E-06	S-adenosylmethioninamine biosynthetic process
BP	GO:0046499	8.28E-09	2.31E-06	S-adenosylmethioninamine metabolic process
MF	GO:0000287	1.91E-08	5.19E-06	magnesium ion binding
BP	GO:0006952	2.58E-08	6.82E-06	defense response
MF	GO:0030246	2.63E-08	6.82E-06	carbohydrate binding
CC	GO:0005886	2.74E-08	6.94E-06	plasma membrane
CC	GO:0009505	6.50E-08	1.61E-05	plant-type cell wall
BP	GO:0006597	7.76E-08	1.86E-05	spermine biosynthetic process
BP	GO:0008295	7.86E-08	1.86E-05	spermidine biosynthetic process
BP	GO:0006468	1.37E-07	3.19E-05	protein phosphorylation
BP	GO:0035556	1.44E-07	3.26E-05	intracellular signal transduction
BP	GO:0008216	1.66E-07	3.70E-05	spermidine metabolic process
BP	GO:0009605	1.94E-07	4.23E-05	response to external stimulus
MF	GO:0004674	2.37E-07	5.06E-05	protein serine/threonine kinase activity
MF	GO:0004672	2.53E-07	5.30E-05	protein kinase activity
CC	GO:0005618	2.75E-07	5.56E-05	cell wall
CC	GO:0030312	2.75E-07	5.56E-05	external encapsulating structure
BP	GO:0009719	3.01E-07	5.96E-05	response to endogenous stimulus
NA	GO:0098869	4.57E-07	8.90E-05	NA
BP	GO:0008215	4.89E-07	9.34E-05	spermine metabolic process
MF	GO:0004022	5.15E-07	9.68E-05	alcohol dehydrogenase (NAD) activity
BP	GO:0006596	5.24E-07	9.68E-05	polyamine biosynthetic process
BP	GO:0005975	6.11E-07	0.000111	carbohydrate metabolic process
BP	GO:0009751	6.73E-07	0.00012	response to salicylic acid
MF	GO:0000155	9.56E-07	0.000166	phosphorelay sensor kinase activity
MF	GO:0016773	9.60E-07	0.000166	phosphotransferase activity, alcohol group as acceptor
BP	GO:0009664	9.82E-07	0.000167	plant-type cell wall organization
BP	GO:0009607	1.01E-06	0.00017	response to biotic stimulus
MF	GO:0004564	1.07E-06	0.000173	beta-fructofuranosidase activity
MF	GO:0004575	1.07E-06	0.000173	sucrose alpha-glucosidase activity
MF	GO:0009815	1.21E-06	0.000189	1-aminocyclopropane-1-carboxylate oxidase activity
MF	GO:0004673	1.21E-06	0.000189	protein histidine kinase activity
MF	GO:0016775	1.21E-06	0.000189	phosphotransferase activity, nitrogenous group as acceptor
BP	GO:0016310	1.39E-06	0.000214	phosphorylation
BP	GO:0071396	1.58E-06	0.000238	cellular response to lipid
BP	GO:0009725	1.79E-06	0.000267	response to hormone
BP	GO:0097305	1.86E-06	0.000275	response to alcohol
BP	GO:0033993	2.22E-06	0.000323	response to lipid
BP	GO:0008152	2.47E-06	0.000354	metabolic process

BP	GO:0010035	2.68E-06	0.000379	response to inorganic substance
BP	GO:0071215	3.97E-06	0.000555	cellular response to abscisic acid stimulus
MF	GO:0003824	4.74E-06	0.000654	catalytic activity
MF	GO:0090599	4.91E-06	0.000668	alpha-glucosidase activity
BP	GO:0014070	5.06E-06	0.000681	response to organic cyclic compound
CC	GO:0016021	6.11E-06	0.000812	integral component of membrane
BP	GO:0009737	6.79E-06	0.000891	response to abscisic acid
CC	GO:0031224	7.39E-06	0.000958	intrinsic component of membrane
BP	GO:0097306	7.60E-06	0.000974	cellular response to alcohol
BP	GO:0016052	7.84E-06	0.000993	carbohydrate catabolic process
MF	GO:0004807	8.07E-06	0.001011	triose-phosphate isomerase activity
MF	GO:0016798	9.08E-06	0.001124	hydrolase activity, acting on glycosyl bonds
BP	GO:0051707	9.28E-06	0.001136	response to other organism
MF	GO:0031418	9.85E-06	0.001193	L-ascorbic acid binding
MF	GO:0016301	1.07E-05	0.001276	kinase activity
MF	GO:0004553	1.25E-05	0.00148	hydrolase activity, hydrolyzing O-glycosyl compounds
MF	GO:0050662	1.38E-05	0.001612	coenzyme binding
MF	GO:0043565	1.41E-05	0.001633	sequence-specific DNA binding
BP	GO:0009785	1.74E-05	0.001992	blue light signaling pathway
BP	GO:0043207	1.99E-05	0.002264	response to external biotic stimulus
BP	GO:0009734	2.14E-05	0.002408	auxin-activated signaling pathway
MF	GO:0008553	2.39E-05	0.002662	hydrogen-exporting ATPase activity, phosphorylative mechanism
BP	GO:0009962	2.43E-05	0.002671	regulation of flavonoid biosynthetic process
BP	GO:0010363	2.45E-05	0.002671	regulation of plant-type hypersensitive response
BP	GO:0071214	2.80E-05	0.003025	cellular response to abiotic stimulus
BP	GO:0006631	2.84E-05	0.003039	fatty acid metabolic process
BP	GO:0031668	3.34E-05	0.003529	cellular response to extracellular stimulus
BP	GO:0032780	3.56E-05	0.003734	negative regulation of ATPase activity
BP	GO:0009723	4.03E-05	0.004127	response to ethylene
BP	GO:0010135	4.07E-05	0.004127	ureide metabolic process
BP	GO:0010136	4.07E-05	0.004127	ureide catabolic process
BP	GO:0009963	4.09E-05	0.004127	positive regulation of flavonoid biosynthetic process
BP	GO:0030522	4.33E-05	0.004325	intracellular receptor signaling pathway
MF	GO:0048037	4.46E-05	0.004424	cofactor binding
BP	GO:0071483	5.25E-05	0.005151	cellular response to blue light
BP	GO:0043562	5.36E-05	0.005218	cellular response to nitrogen levels
BP	GO:0019740	5.44E-05	0.005249	nitrogen utilization
BP	GO:0097164	5.54E-05	0.005267	ammonium ion metabolic process
BP	GO:0006796	5.56E-05	0.005267	phosphate-containing compound metabolic process
BP	GO:0042455	5.83E-05	0.005475	ribonucleoside biosynthetic process

BP	GO:0071472	6.30E-05	0.005864	cellular response to salt stress
BP	GO:0032870	6.41E-05	0.005916	cellular response to hormone stimulus
BP	GO:0009163	6.60E-05	0.006043	nucleoside biosynthetic process
BP	GO:0042451	6.84E-05	0.006104	purine nucleoside biosynthetic process
BP	GO:0046129	6.84E-05	0.006104	purine ribonucleoside biosynthetic process
MF	GO:0000062	6.86E-05	0.006104	fatty-acyl-CoA binding
MF	GO:0042030	6.89E-05	0.006104	ATPase inhibitor activity
BP	GO:0006096	6.98E-05	0.006133	glycolytic process
BP	GO:0006635	7.29E-05	0.006306	fatty acid beta-oxidation
BP	GO:0019395	7.29E-05	0.006306	fatty acid oxidation
BP	GO:0042742	8.47E-05	0.00727	defense response to bacterium
MF	GO:0004872	9.11E-05	0.007704	receptor activity
BP	GO:0098661	9.12E-05	0.007704	inorganic anion transmembrane transport
BP	GO:0009416	9.41E-05	0.007885	response to light stimulus
BP	GO:0009692	0.000108	0.008752	ethylene metabolic process
BP	GO:0009693	0.000108	0.008752	ethylene biosynthetic process
BP	GO:0043449	0.000108	0.008752	cellular alkene metabolic process
BP	GO:0043450	0.000108	0.008752	alkene biosynthetic process
BP	GO:1900674	0.000108	0.008752	olefin biosynthetic process
MF	GO:0052638	0.000113	0.009004	indole-3-butyrate beta-glucosyltransferase activity
BP	GO:0006793	0.000113	0.009004	phosphorus metabolic process
BP	GO:0009062	0.000116	0.00916	fatty acid catabolic process
BP	GO:0000256	0.000125	0.009787	allantoin catabolic process
MF	GO:0003995	0.000131	0.010041	acyl-CoA dehydrogenase activity
BP	GO:0033539	0.000131	0.010041	fatty acid beta-oxidation using acyl-CoA dehydrogenase
MF	GO:0052890	0.000131	0.010041	oxidoreductase activity, acting on the CH-CH group of donors, with a flavin as acceptor
MF	GO:0016641	0.000132	0.010041	oxidoreductase activity, acting on the CH-NH2 group of donors, oxygen as acceptor
BP	GO:0072488	0.000137	0.010379	ammonium transmembrane transport
BP	GO:0006094	0.00014	0.010474	gluconeogenesis
BP	GO:0019319	0.00014	0.010474	hexose biosynthetic process
BP	GO:0042538	0.000149	0.011074	hyperosmotic salinity response
MF	GO:0016813	0.000152	0.011219	hydrolase activity, acting on carbon-nitrogen (but not peptide) bonds, in linear amidines
BP	GO:0008150	0.000156	0.011431	biological_process
MF	GO:0015926	0.000169	0.012288	glucosidase activity
BP	GO:2000762	0.00018	0.012957	regulation of phenylpropanoid metabolic process
MF	GO:0047652	0.000181	0.012972	allantoate deiminase activity
MF	GO:0043167	0.000183	0.013069	ion binding
MF	GO:0043169	0.000187	0.01318	cation binding
BP	GO:0009753	0.000187	0.01318	response to jasmonic acid

BP	GO:0055088	0.000195	0.013619	lipid homeostasis
BP	GO:0009269	0.000205	0.014232	response to desiccation
BP	GO:0032787	0.000213	0.014572	monocarboxylic acid metabolic process
BP	GO:0080024	0.000213	0.014572	indolebutyric acid metabolic process
BP	GO:0071496	0.000217	0.01475	cellular response to external stimulus
BP	GO:0046364	0.000219	0.014799	monosaccharide biosynthetic process
BP	GO:0043605	0.000222	0.014951	cellular amide catabolic process
BP	GO:0009744	0.000224	0.014954	response to sucrose
MF	GO:0051213	0.000239	0.015862	dioxygenase activity
BP	GO:0034440	0.000268	0.017708	lipid oxidation
BP	GO:0009617	0.000271	0.017814	response to bacterium
BP	GO:0098542	0.000288	0.018728	defense response to other organism
BP	GO:0006351	0.00029	0.018728	transcription, DNA-templated
BP	GO:0097659	0.00029	0.018728	nucleic acid-templated transcription
MF	GO:0003997	0.000311	0.019925	acyl-CoA oxidase activity
BP	GO:1900673	0.000316	0.020131	olefin metabolic process
MF	GO:0008519	0.000325	0.020502	ammonium transmembrane transporter activity
MF	GO:0015101	0.000325	0.020502	organic cation transmembrane transporter activity
BP	GO:0009628	0.000328	0.020522	response to abiotic stimulus
MF	GO:0060089	0.000333	0.020743	molecular transducer activity
BP	GO:0080163	0.000345	0.021307	regulation of protein serine/threonine phosphatase activity
MF	GO:0046872	0.000348	0.021307	metal ion binding
BP	GO:0071470	0.000349	0.021307	cellular response to osmotic stress
BP	GO:0071475	0.00035	0.021307	cellular hyperosmotic salinity response
BP	GO:0016042	0.00038	0.023022	lipid catabolic process
BP	GO:0006972	0.000393	0.023649	hyperosmotic response
BP	GO:0070887	0.000395	0.023658	cellular response to chemical stimulus
BP	GO:0034285	0.000399	0.023768	response to disaccharide
BP	GO:0071310	0.000401	0.023768	cellular response to organic substance
MF	GO:0004857	0.000422	0.024881	enzyme inhibitor activity
BP	GO:0009314	0.000442	0.025891	response to radiation
MF	GO:0016638	0.000444	0.025891	oxidoreductase activity, acting on the CH-NH2 group of donors
BP	GO:0042592	0.000448	0.025998	homeostatic process
CC	GO:0044425	0.000458	0.026332	membrane part
BP	GO:0006995	0.000459	0.026332	cellular response to nitrogen starvation
BP	GO:0034654	0.000471	0.026848	nucleobase-containing compound biosynthetic process
BP	GO:0098662	0.000481	0.027134	inorganic cation transmembrane transport
MF	GO:0016634	0.000481	0.027134	oxidoreductase activity, acting on the CH-CH group of donors, oxygen as acceptor
BP	GO:0072329	0.00049	0.027501	monocarboxylic acid catabolic process
MF	GO:0033897	0.000496	0.027712	ribonuclease T2 activity

MF	GO:0052739	0.000512	0.028229	phosphatidylserine 1-acylhydrolase activity
MF	GO:0052740	0.000512	0.028229	1-acyl-2-lysophosphatidylserine acylhydrolase activity
MF	GO:0001228	0.000513	0.028229	transcriptional activator activity, RNA polymerase II transcription regulatory region sequence-specific binding
BP	GO:0015696	0.000523	0.028656	ammonium transport
BP	GO:0006970	0.000534	0.028895	response to osmotic stress
BP	GO:0006787	0.000541	0.028895	porphyrin-containing compound catabolic process
BP	GO:0015996	0.000541	0.028895	chlorophyll catabolic process
BP	GO:0033015	0.000541	0.028895	tetrapyrrole catabolic process
BP	GO:0046149	0.000541	0.028895	pigment catabolic process
BP	GO:0009651	0.000554	0.029439	response to salt stress
MF	GO:0038023	0.000572	0.03025	signaling receptor activity
MF	GO:0010427	0.000582	0.030561	abscisic acid binding
BP	GO:0009750	0.000583	0.030561	response to fructose
MF	GO:0042562	0.000586	0.030576	hormone binding
BP	GO:0009309	0.000599	0.030853	amine biosynthetic process
BP	GO:0042401	0.000599	0.030853	cellular biogenic amine biosynthetic process
BP	GO:0098660	0.0006	0.030853	inorganic ion transmembrane transport
CC	GO:0005783	0.000628	0.032151	endoplasmic reticulum
BP	GO:0044724	0.000641	0.032666	single-organism carbohydrate catabolic process
MF	GO:0004620	0.000666	0.033779	phospholipase activity
BP	GO:0071495	0.000699	0.035249	cellular response to endogenous stimulus
MF	GO:0003674	0.000714	0.03572	molecular_function
BP	GO:0009414	0.000715	0.03572	response to water deprivation
MF	GO:0032441	0.000727	0.036167	pheophorbide a oxygenase activity
BP	GO:0042445	0.000748	0.037062	hormone metabolic process
BP	GO:0009743	0.000776	0.038283	response to carbohydrate
BP	GO:0046128	0.000781	0.038356	purine ribonucleoside metabolic process
BP	GO:0006595	0.000789	0.038581	polyamine metabolic process
BP	GO:0071474	0.000798	0.038843	cellular hyperosmotic response
BP	GO:0031669	0.000816	0.039504	cellular response to nutrient levels
BP	GO:0009620	0.00085	0.040732	response to fungus
MF	GO:0016892	0.000852	0.040732	endoribonuclease activity, producing 3'-phosphomonoesters
MF	GO:0016894	0.000852	0.040732	endonuclease activity, active with either ribo- or deoxyribonucleic acids and producing 3'-phosphomonoesters
BP	GO:0007154	0.000856	0.040732	cell communication
MF	GO:0000981	0.000887	0.042022	RNA polymerase II transcription factor activity, sequence-specific DNA binding
MF	GO:0043178	0.000895	0.042232	alcohol binding
BP	GO:0009119	0.000922	0.043292	ribonucleoside metabolic process
MF	GO:0019840	0.000927	0.04334	isoprenoid binding

BP	GO:0000255	0.000937	0.043628	allantoin metabolic process
BP	GO:0048878	0.000956	0.044322	chemical homeostasis
BP	GO:0006865	0.000963	0.044451	amino acid transport
BP	GO:0010817	0.000979	0.04501	regulation of hormone levels
MF	GO:0048029	0.001	0.045785	monosaccharide binding
BP	GO:0061615	0.001025	0.046748	glycolytic process through fructose-6-phosphate
BP	GO:0009718	0.001042	0.047316	anthocyanin-containing compound biosynthetic process
BP	GO:0009733	0.001052	0.047556	response to auxin
BP	GO:0043462	0.001093	0.049201	regulation of ATPase activity
